# Inhibition of ceramide accumulation in *AdipoR1^–/–^* mice increases photoreceptor survival and improves vision

**DOI:** 10.1172/jci.insight.156301

**Published:** 2022-02-22

**Authors:** Dominik Lewandowski, Andrzej T. Foik, Roman Smidak, Elliot H. Choi, Jianye Zhang, Thanh Hoang, Aleksander Tworak, Susie Suh, Henri Leinonen, Zhiqian Dong, Antonio F.M. Pinto, Emily Tom, Jennings Luu, Joan Lee, Xiuli Ma, Erhard Bieberich, Seth Blackshaw, Alan Saghatelian, David C. Lyon, Dorota Skowronska-Krawczyk, Marcin Tabaka, Krzysztof Palczewski

**Affiliations:** 1 Department of Ophthalmology, Gavin Herbert Eye Institute, UCI, Irvine, California, USA.; 2 International Center for Translational Eye Research, Institute of Physical Chemistry, Polish Academy of Sciences, Warsaw, Poland.; 3 Medical Scientist Training Program, Case Western Reserve University, Cleveland, Ohio, USA.; 4 Solomon H. Snyder Department of Neuroscience, Johns Hopkins University, Baltimore, Maryland, USA.; 5 School of Pharmacy, University of Eastern Finland, Kuopio, Finland.; 6 Polgenix Inc., Department of Medical Devices, Cleveland Ohio, USA.; 7 Clayton Foundation Laboratories for Peptide Biology, Salk Institute for Biological Studies, La Jolla, California, USA.; 8 MetroHealth Medical Center, Case Western Reserve University, Cleveland, Ohio, USA.; 9 Department of Physiology, University of Kentucky, Lexington, Kentucky, USA.; 10 Department of Anatomy and Neurobiology,; 11 Department of Physiology and Biophysics,; 12 Department of Chemistry, and; 13 Department of Molecular Biology and Biochemistry, UCI, Irvine, California, USA.

**Keywords:** Ophthalmology, Drug therapy

## Abstract

Adiponectin receptor 1 (ADIPOR1) is a lipid and glucose metabolism regulator that possesses intrinsic ceramidase activity. Mutations of the *ADIPOR1* gene have been associated with nonsyndromic and syndromic retinitis pigmentosa. Here, we show that the absence of *AdipoR1* in mice leads to progressive photoreceptor degeneration, significant reduction of electroretinogram amplitudes, decreased retinoid content in the retina, and reduced cone opsin expression. Single-cell RNA-Seq results indicate that *ADIPOR1* encoded the most abundantly expressed ceramidase in mice and one of the 2 most highly expressed ceramidases in the human retina, next to acid ceramidase *ASAH1*. We discovered an accumulation of ceramides in the *AdipoR1^–/–^* retina, likely due to insufficient ceramidase activity for healthy retina function, resulting in photoreceptor death. Combined treatment with desipramine/L-cycloserine (DC) lowered ceramide levels and exerted a protective effect on photoreceptors in *AdipoR1^–/–^* mice. Moreover, we observed improvement in cone-mediated retinal function in the DC-treated animals. Lastly, we found that prolonged DC treatment corrected the electrical responses of the primary visual cortex to visual stimuli, approaching near-normal levels for some parameters. These results highlight the importance of ADIPOR1 ceramidase in the retina and show that pharmacological inhibition of ceramide generation can provide a therapeutic strategy for *ADIPOR1-*related retinopathy.

## Graphical abstract

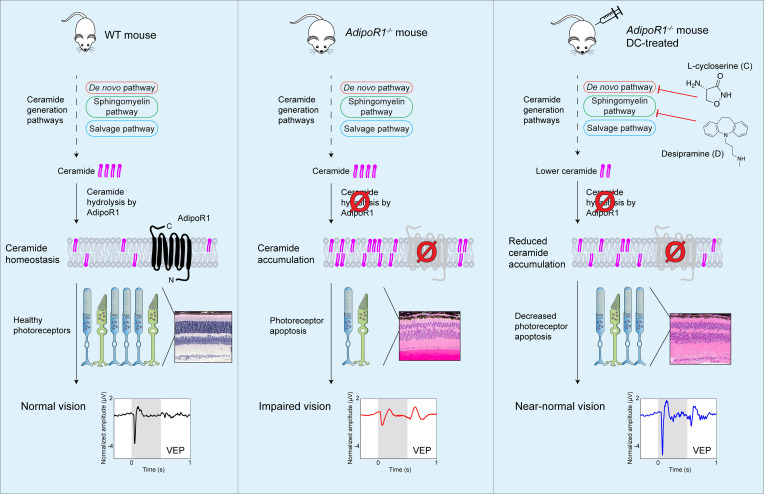

## Introduction

*ADIPOR1* encodes the 7-transmembrane adiponectin receptor 1 (ADIPOR1), which mediates metabolic actions of adiponectin, such as insulin-sensitizing, antiinflammatory, and antiapoptotic functions (1). ADIPOR1 is engaged in lipid and glucose metabolism by activating AMPK pathways (2, 3). ADIPOR1 was also shown to regulate membrane viscosity in an adiponectin-independent manner (4). Although the *ADIPOR1* gene is ubiquitously expressed in multiple organs, the highest ADIPOR1 protein levels are found in the eye and brain, suggesting its critical importance in these neural tissues (5), and *ADIPOR1* expression is enriched in photoreceptors relative to other retinal cell types (6). In fact, *ADIPOR1* is one of many genes implicated in retinal degeneration. Rice and colleagues have shown that *AdipoR1* is essential for healthy retinal function, where it preferentially promotes accumulation of docosahexaenoic acid (DHA) over other essential fatty acids in the retina, supporting selective enrichment of DHA in the photoreceptor cells in mice (7). Mutations of *ADIPOR1* in humans have been associated with nonsyndromic retinitis pigmentosa (RP) and syndromic RP combined with intellectual disability (8, 9). Furthermore, SNP in *ADIPOR1* was identified as a genetic risk factor for advanced age-related macular degeneration (AMD) in the Finnish population (10). ADIPOR1 was also demonstrated to possess intrinsic ceramidase activity, which can be further stimulated upon binding adiponectin (1, 11). Nevertheless, adiponectin-KO mice did not show retinal degeneration, suggesting that essential functions of ADIPOR1 in the eye are independent of adiponectin (7).

Ceramides are sphingolipids essential for eukaryotic cell membrane stability and act as potent signaling molecules in inflammation, cell cycle arrest, cell death, and heat shock response pathways. Imbalance in the ceramide profiles and elevated levels of ceramides have been found in diseases such as cancer, Alzheimer’s disease, type 2 diabetes, multiple sclerosis, cardiovascular disease, and nonalcoholic fatty liver disease; for this reason, they have recently been proposed as promising biomarker candidates (12–14). Ceramide dyshomeostasis is detrimental for the retina, increasing inflammatory events and unsettling the balance of cell death or survival (15, 16).

In this study, we discovered that ADIPOR1 is the most abundantly expressed ceramidase in the mouse eye, and its ablation in *AdipoR1-*KO mice leads to insufficient ceramidase activity for healthy retina function, resulting in ceramide accumulation and photoreceptor degeneration. We also devised a pharmacological treatment to alleviate the detrimental effects of ceramide accumulation in the retina to preserve the structure and function of this light-sensitive tissue.

## Results

### Structural and functional characterization of the retina of AdipoR1^–/–^ mice.

**Figure 1:**
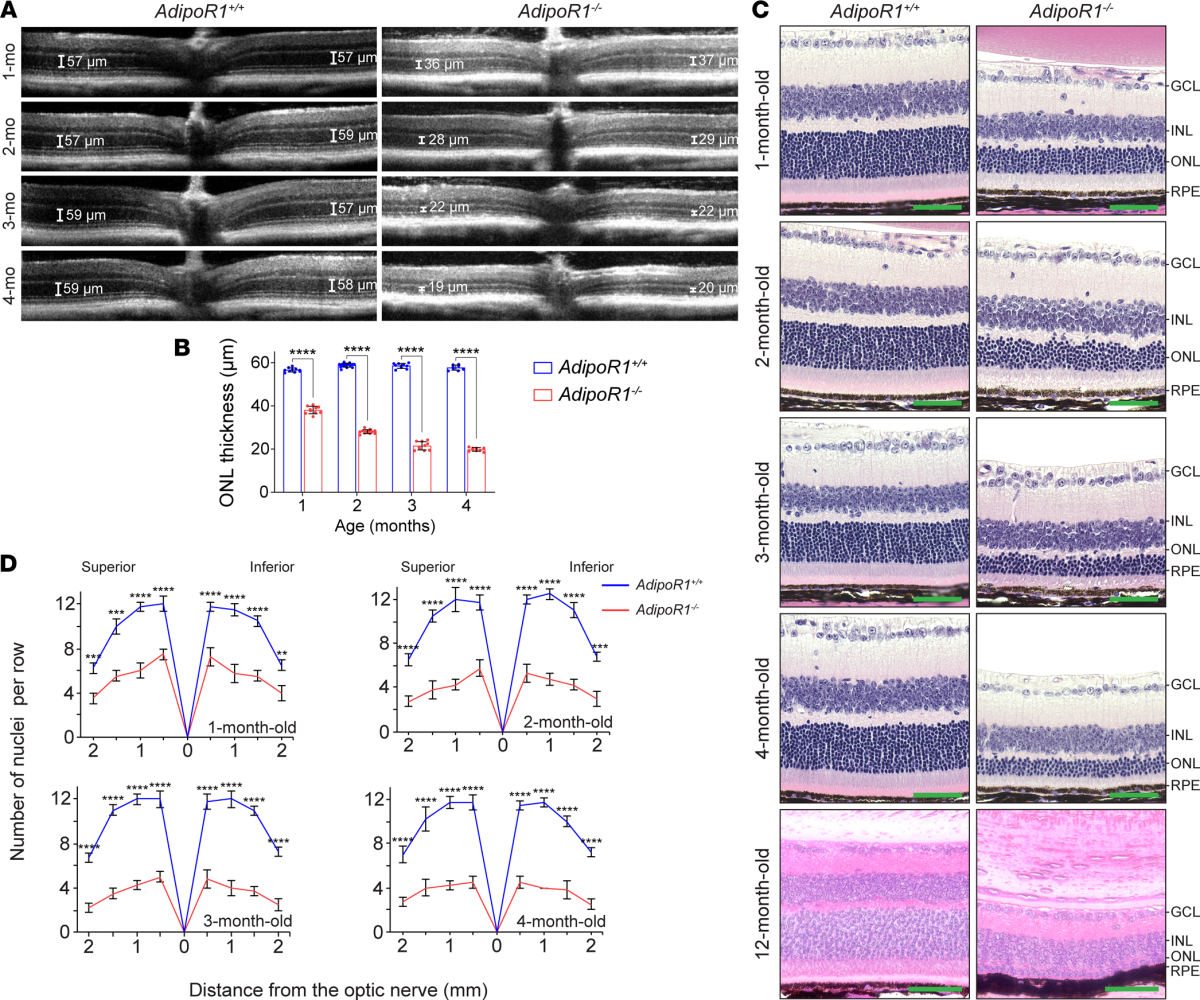
Structural characterization of the retinas in *AdipoR1^–/–^* mice. (**A**) Representative OCT images of retinas from 1- to 4-month-old *AdipoR1*^–/–^ and *AdipoR1^+/+^* mice, showing loss of thickness of the ONL in the KO mice. The scale bar indicates the thickness of the ONL measured 500 μm from the optic nerve head (ONH). (**B**) The average ONL thicknesses were measured from the OCT images (*n* ≥ 8 eyes). (**C**) H&E-stained sections of retinas from *AdipoR1^+/+^* and *AdipoR1^–/–^* mice at different ages, demonstrating progressive degeneration of photoreceptors in the KO animals. Images were taken 500–750 μm from the ONH. Scale bar: 50 μm. (**D**) The number of photoreceptor nuclei at increasing distances from the ONH expressed as spider plots; *n* = 4 for each genotype and age. GCL, ganglion cell layer; INL, inner nuclear layer; ONL, outer nuclear layer; RPE, retinal pigmented epithelium. In **B** and **D**, data are shown as mean ± SD, and statistical significance was determined by a 2-tailed Student’s *t* test; **P < 0.01, ***P < 0.001, ****P < 0.0001.

We first evaluated the structure of the retina in *AdipoR1^–/–^* mice. Optical coherence tomography (OCT) of the retina of *AdipoR1^–/–^* mice ranging from 1 to 4 months of age revealed gradual thinning of the outer nuclear layer (ONL). In 1-month-old *AdipoR1^–/–^* mice, there was about a 33% decrease in ONL thickness compared with WT. With age, thinning of the ONL in the KO mice progressed to ~28 μm in 2-month-old (52% decrease), ~22 μm in 3-month-old (63% decrease), and ~20 μm (66% decrease) in 4-month-old mice (Figure 1, A and B).

The OCT results were confirmed by histology, which showed progressive photoreceptor degeneration. In WT 1- to 4-month-old animals, the number of nuclei in the ONL calculated at 500–1000 μm from the optic nerve was stable and averaged about 11–12 nuclei. In contrast, in *AdipoR1^–/–^* mice, the number of ONL nuclei was significantly decreased to 7–8 in 1-month-old animals, and around 6, 5, and 4 in 2-, 3-, and 4-month-old KO mice, respectively (Figure 1, C and D). In 12-month-old *AdipoR1^–/–^* mice, only 1 row of photoreceptor nuclei remained (Figure 1C). Moreover, the histologic data reveal gradual shortening of the photoreceptor inner and outer segments in *AdipoR1^–/–^* mice, further reflecting photoreceptor structural degeneration (Figure 1C).

**Figure 2:**
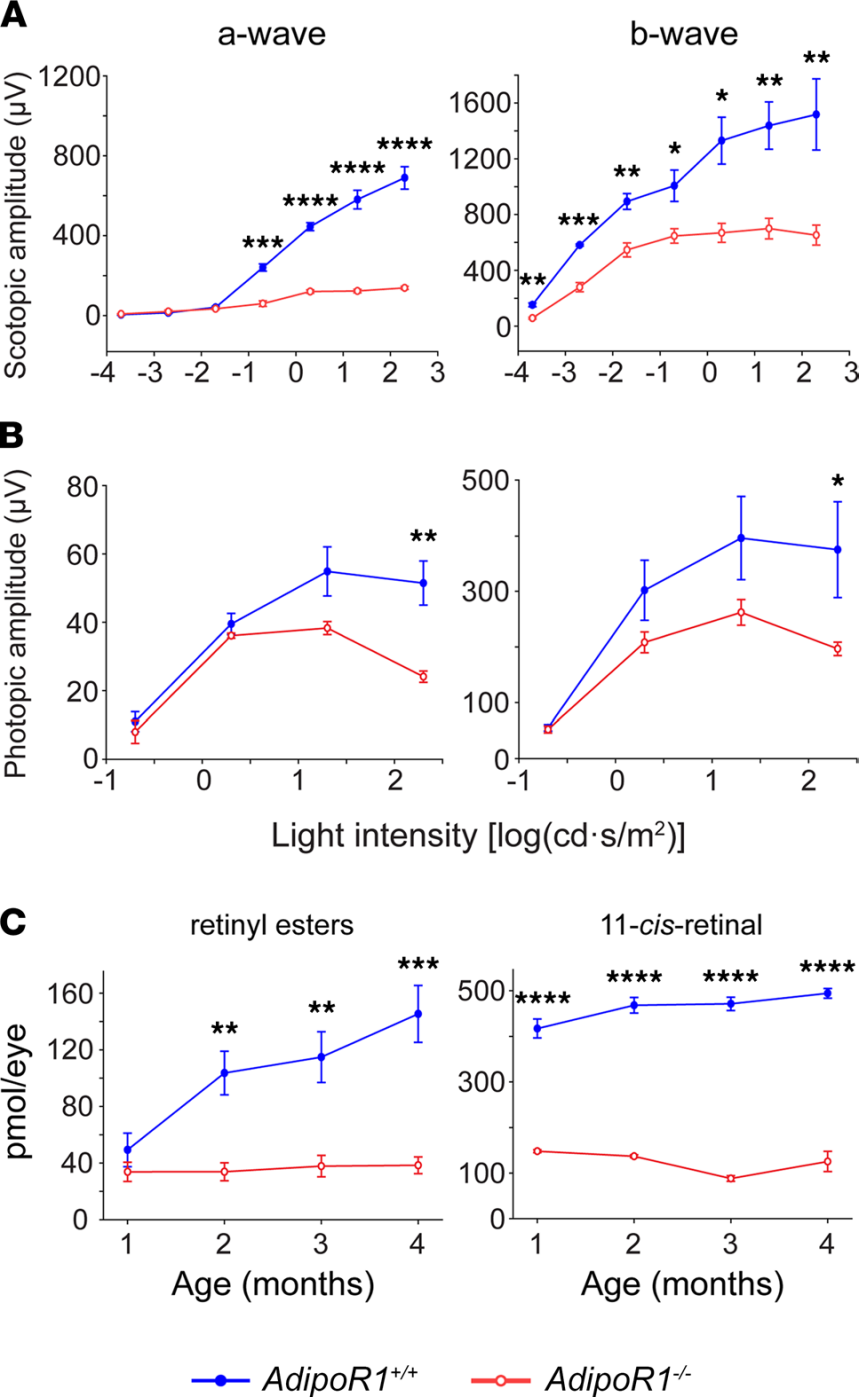
Functional characterization of the retinas in *AdipoR1^–/–^* mice. (**A** and **B**) Serial ERG responses to increasing flash stimuli were obtained for selected intensities under dark-adapted and light-adapted conditions in 1-month-old *AdipoR1^–/–^* and WT mice. (**A**) Scotopic a-wave and b-wave amplitudes were dramatically attenuated in *AdipoR1^–/–^* mice. (**B**) Photopic a-wave and b-wave amplitudes. Data are shown as mean ± SEM; *n* = 4 for all genotypes and ages. (**C**) Greatly reduced levels of retinoids: 11-*cis*-retinal and retinyl esters, indicating an impaired visual cycle in *AdipoR1^–/–^* mice. Retinoids were quantified by HPLC analysis (*n* = 4 for each age and genotype). All data are shown as mean ± SD. In **A** and **B**, statistical significance was determined with repeated-measures 2-way ANOVA followed by Sidak’s post hoc test; in **C**, a 2-tailed Student’s *t* test was used; *P < 0.05, **P < 0.01, ***P < 0.001, ****P < 0.0001.

To evaluate the physiological function of the retina, we performed electroretinography (ERG) recordings in 1- to 4-month-old *AdipoR1^–/–^* and WT mice. Scotopic responses from rods and cones of dark-adapted animals showed a severe attenuation in a-wave amplitudes in KO mice, indicating low to almost no response from photoreceptors in all age groups of KO animals (Figure 2A and Supplemental Figure 1A; supplemental material available online with this article; https://doi.org/10.1172/jci.insight.156301DS1). Scotopic ERG recordings of the b-wave amplitudes (arising primarily from depolarizing bipolar cells) also showed significant differences between 1-month-old *AdipoR1*-KO and WT mice (Figure 2A). For 2-, 3-, and 4-month-old *AdipoR1^–/–^* mice, there were significant differences at higher light intensities when compared with WT (Supplemental Figure 1B). Photopic a-wave amplitudes were also significantly reduced, showing impaired cone functions in all age groups (Figure 2B and Supplemental Figure 1C). The cone responses were already considerably weaker at the highest light intensities in 1-month-old *AdipoR1^–/–^* mice. The responses continued to decrease with age so that the 4-month-old KO mice displayed very weak responses to all light intensities (Supplemental Figure 1C). Examination of photopic b-wave amplitudes showed a significant decrease only with 1-month-old KO mice at the highest light intensity stimulus (Figure 2B and Supplemental Figure 1D).

To evaluate the visual cycle activity in *AdipoR1^–/–^* mice, retinoid levels were quantified in the eyes of dark-adapted animals. HPLC analysis indicated that 11-*cis*-retinal contents gradually decreased as the *AdipoR1^–/–^* mice aged and were approximately 3- to 5-fold lower in the KO mice between 1- and 4-months-old compared with WT (Figure 2C). Retinyl ester levels were not significantly different in 1-month-old *AdipoR1^–/–^* versus WT mice, but the levels were decreased about 3- to 4-fold in 2-, 3-, and 4-month-old KO mice (Figure 2C and Supplemental Figure 3A). These results indicate that the retinoid visual cycle is severely impaired in *AdipoR1^–/–^* mice for all 4 age groups.

Moreover, to explore further changes in the retinoid cycle in the absence of its key elements, we profiled retinoids in the *AdipoR1^–/–^ Rpe65^–/–^* and *AdipoR1^–/–^ Abca4^–/–^* mice (Supplemental Figure 3A). The retinyl ester analysis showed no differences in *AdipoR1^–/–^ Rpe65^–/–^* and *AdipoR1^–/–^ Abca4^–/–^* mice when compared with *AdipoR1^–/–^* mice; and 11-*cis*-retinal was at the same level in *AdipoR1^–/–^* and *AdipoR1^–/–^ Abca4^–/–^* mice, but as expected, it was not detected in *AdipoR1^–/–^ Rpe65^–/–^* double KO mice.

To examine whether the lack of ADIPOR1 results in an accumulation of toxic metabolites coming from the retinoid cycle, we additionally evaluated *bis*-retinoid A2E (N-retinylidene-N-retinylethanolamine) levels in mice with different genotypes. A2E quantity was similar in *AdipoR1^–/–^ Abca4^–/–^* mice compared with WT and below the detection limit in *AdipoR1^–/–^ Rpe65^–/–^* mice. For all of the examined genotypes, A2E was much lower than reported for *Abca4^–/–^ Rdh8^–/–^* mice, which typically accumulate large amounts of A2E in their retinal pigment epithelium (RPE) cells (Supplemental Figure 3B) (17). This result suggests that the lack of the *AdipoR1* gene does not affect the clearance of all-*trans*-retinal.

**Figure 3:**
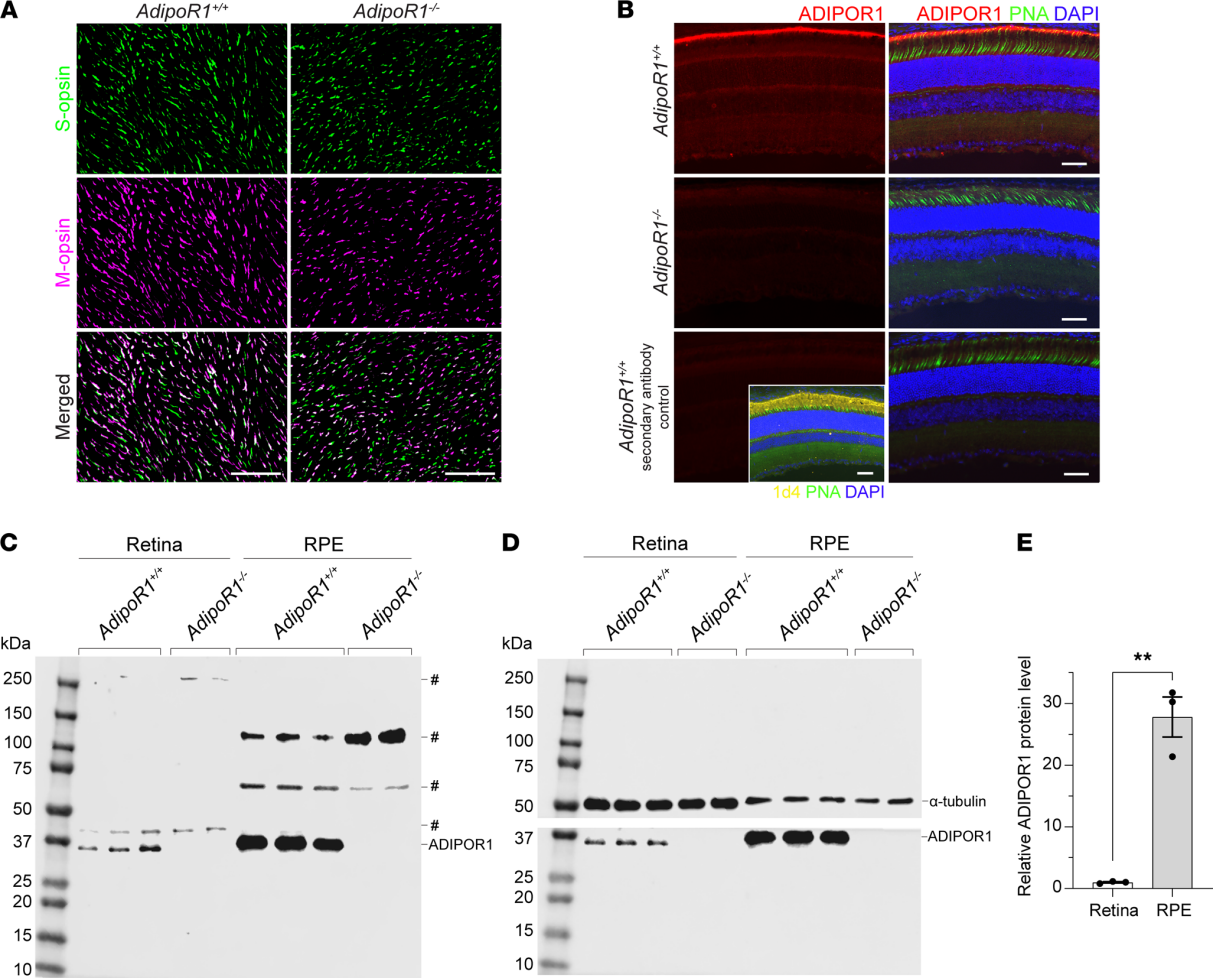
Expression of ADIPOR1 protein and cone opsins in 1-month-old *AdipoR1^–/–^* and WT mouse retinas. (**A**) Representative S-opsin and M-opsin staining of cones on retinal flatmounts from *AdipoR1^+/+^* and *AdipoR1^–/–^* mice. Images were collected from the ventral/nasal quadrant at 500–750 μm from the ONH. Scale bar: 50 μm. (**B**) IHC staining of a mouse retinal cross section with ADIPOR1 Ab (red) indicated the strongest ADIPOR1 expression at the interface between the RPE apical microvilli and the distal part of the photoreceptor OS. Cones were stained with PNA-fluorescein (green), and cell nuclei were stained with DAPI (blue). Inset on the lower-left panel of **B** shows rhodopsin staining with 1d4 Ab to confirm the integrity of the photoreceptor-RPE interface on the retinal cross sections used in the experiment. Scale bars: 20 µm. (**C** and **D**) Western blots of mouse neural retinas or RPE cells from 1-month-old *AdipoR1^–/–^* and WT mice, stained with ADIPOR1 Ab. Each lane represents the sample from an individual mouse, for which an equal amount of total protein was loaded. Nonspecific bands are labeled with #. (**D**) α-Tubulin Ab was used as a loading control (*n* = 3 for WT, *n* = 2 for KO). (**E**) Densitometric quantification of Western blots in **D**, normalized to α-tubulin. Statistical significance was determined by a 2-tailed Student’s *t* test; **P < 0.01.

To investigate if cone opsin expression is affected by *AdipoR1* KO, the whole retina was flat-mounted and stained with S-opsin and M-opsin Abs. Both S-cone and M-cone opsins were detected in the retina of 1-month-old KO mice, and their distribution within all 4 retinal quadrants was similar to WT. However, a decrease in both S-opsin and M-opsin expression was observed at higher magnification in the ventral/nasal quadrant (Figure 3A) compared with the age-matched WT retinas. The diminished opsin-positive punctate fluorescence indicated reduced S/M-opsin expression and suggested that degeneration of cone outer segments occurred already in 1-month-old *AdipoR1^–/–^* mice.

### ADIPOR1 localizes predominantly on the RPE apical processes and photoreceptor tips interface.

To determine the expression levels and localization of the ADIPOR1 protein in the mouse retinas, we stained albino mouse-eye cryosections from 1-month-old WT mice with ADIPOR1 Ab. *AdipoR1*-KO mice were used as a negative control for the Ab specificity. ADIPOR1 was present in the whole retina, but its strongest signal was noticed at the interface between the RPE apical microvilli and the distal part of the photoreceptor outer segments, rather than the basal or middle sections of the outer segments (Figure 3B). This position was confirmed by colocalization (merged image) of the red-stained ADIPOR1 protein with the green-stained cone outer segment tips labeled with peanut agglutinin (PNA) (Figure 3B, upper right). Also, the basolateral RPE membranes or membranes inside the RPE cell showed a much weaker signal for ADIPOR1 than the apical processes. The lowest ADIPOR1 content was noticed in the ONL and inner nuclear layer (INL) (Figure 3B). A very weak to no signal was observed in the retinas of *AdipoR1^–/–^* mice, or in the retinas of *AdipoR1^+/+^* mice stained with the secondary Ab only. Rhodopsin staining was performed to show the preserved interface between rods and the RPE (Figure 3B, inset).

To evaluate the level of ADIPOR1 protein in the RPE cells and the whole neural retina, we performed Western blots (Figure 3C). The analysis showed that ADIPOR1 protein is present in both the retina and RPE cells (Figure 3C). However, the RPE cells had over 27-fold higher ADIPOR1 protein content than the neural retina, as indicated by quantification of band intensities, normalized to α-tubulin (Figure 3, D and E). The specificity of the Ab was confirmed by undetectable immunoreaction for ADIPOR1 protein in the KO animals.

### Microglia/macrophages infiltrate to the subretinal space in AdipoR1^–/–^ mouse eyes.

**Figure 4:**
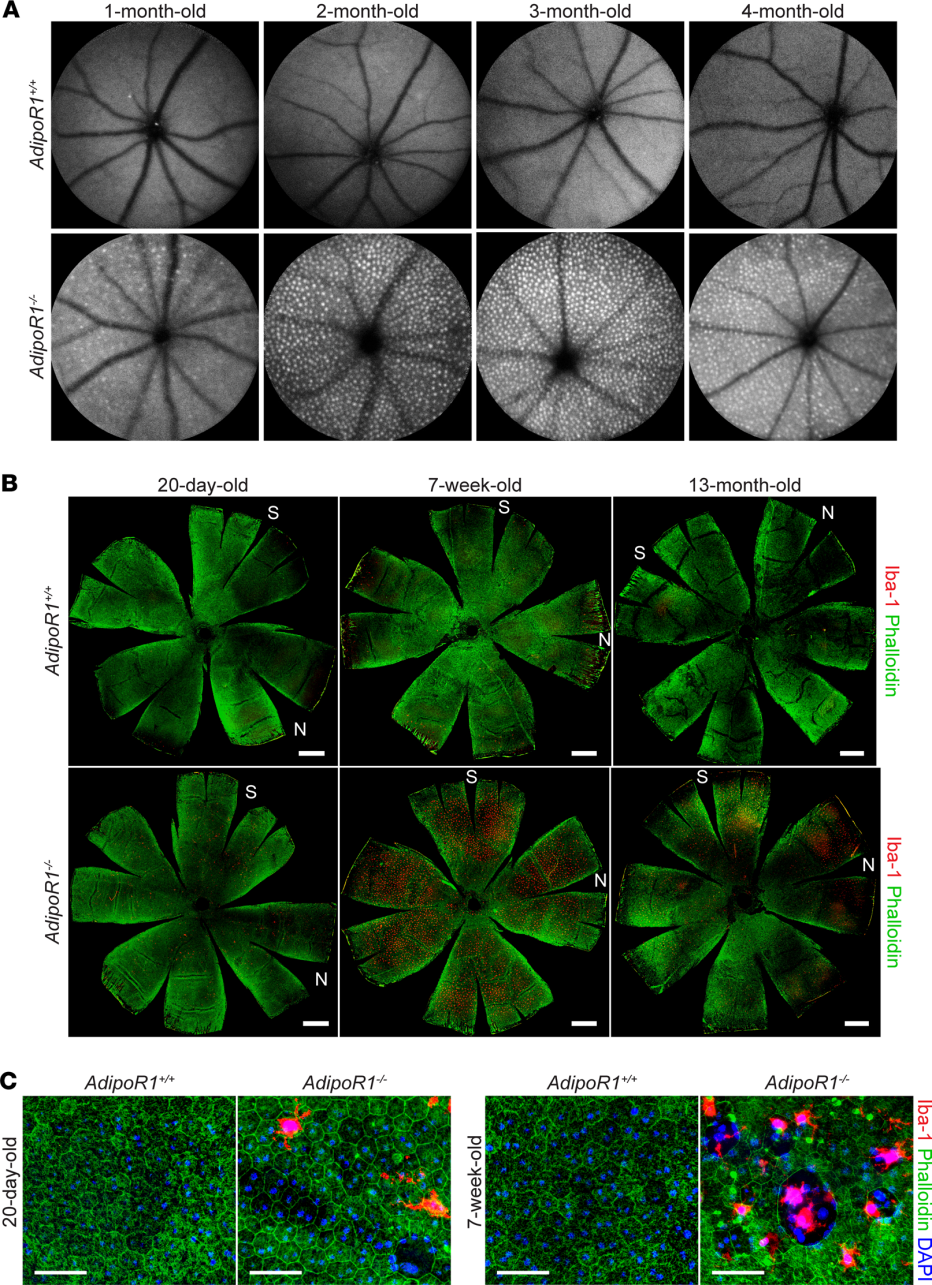
Microglia/macrophage infiltration to the subretinal space in *AdipoR1^–/–^* mice. (**A**) Representative SLO images showing autofluorescent spots that could result from macrophages/microglia infiltrating the subretinal space in the eye fundus of *AdipoR1*^–/–^ mice. (**B**) Representative Iba1 (red) and phalloidin (green) staining on the RPE eyecup flatmounts of *AdipoR1*^–/–^ mice showing a massive influx of macrophage/microglia toward the photoreceptor-RPE interface. S and N represent superior and nasal retinal quadrants, respectively. Scale bar: 500 μm. (**C**) Higher magnification of the RPE wholemount from **B** additionally shows broken epithelial barriers observed throughout the RPE in 7-week-old *AdipoR1*^–/–^ mice. Images were taken from the range of 500 μm to 2000 μm from the ONH. Scale bar: 50 μm.

To further assess changes in the degenerating retina, we performed scanning laser ophthalmoscopy (SLO). In vivo SLO examination revealed accumulation of autofluorescent spots in the eye fundus of 1-month-old *AdipoR1^–/–^* mice (Figure 4A). This accumulation became more evident as the photoreceptor degeneration progressed in 2-, 3-, and 4-month-old KO mice (Figure 4A). These autofluorescent signals have been interpreted previously to indicate phagocytosing microglia/macrophages, which are a sign of ongoing inflammation in mouse models of retinal degeneration (18–20). To detect if microglia/macrophage cells infiltrate subretinal space, an immune-privileged site in a healthy retina, we stained RPE flatmounts from 20-day, 7-week, and 13-month-old *AdipoR1^–/–^* mice with an anti-Iba1 Ab (Figure 4B). Iba1^+^ staining revealed microglia/macrophage cells attached to the RPE layer, already in 20-day-old *AdipoR1^–/–^* mice (Figure 4, B and C), suggesting that the inflammatory events started before the onset of photoreceptor degeneration, previously demonstrated to occur after P20 (7). By 7 weeks of age, massive infiltration of microglia/macrophage cells to the subretinal space was detected (Figure 4B, bottom center), which correlated with the increasing number of autofluorescent spots observed with SLO. In 13-month-old *AdipoR1^–/–^* mice, with approximately 1 row of photoreceptors remaining (Figure 1B), the number of microglia/macrophage cells decreased (Figure 4B, bottom right). In contrast, in the RPE of age-matched WT mice, microglia/macrophages either were not found or were detected as sparsely distributed single cells within the area of 0–2000 μm from the optic nerve (Figure 4B, top panels). Together, these findings confirm an early immune response in the subretinal space of *AdipoR1^–/–^* mice. Higher magnification of the RPE flatmounts showed microglia/macrophages residing in the apical RPE (Figure 4C). Moreover, the formation of numerous pores between RPE cells indicated epithelial barrier breakdown in 7-week-old *AdipoR1^–/–^* mice (Figure 4C, right panels), which most likely allows inflammatory cell traffic from the choroid to the subretinal space (21, 22). Furthermore, we observed the loss of regular hexagonal cellular boundaries in phalloidin-stained flatmounts, as well as enlarged RPE cell dimensions, indicating RPE degeneration already in 20-day-old KO mice. These pathological changes were then much more visible in 7-week-old KO mice. Additionally, in 20-day-old and 7-week-old KO mice, we noticed that the F-actin density from the tight junctions and RPE microvilli was thinner and less abundant than in WT.

### Ceramides accumulate in the retina and RPE of AdipoR1^–/–^ mice.

ADIPOR1 protein possesses an intrinsic ceramidase activity (11). Therefore, we quantified ceramides in the retinas and RPE eyecups of KO and WT animals to determine whether ceramide levels were altered in the *AdipoR1^–/–^* mice due to insufficient ceramidase activity in these tissues.

**Figure 5:**
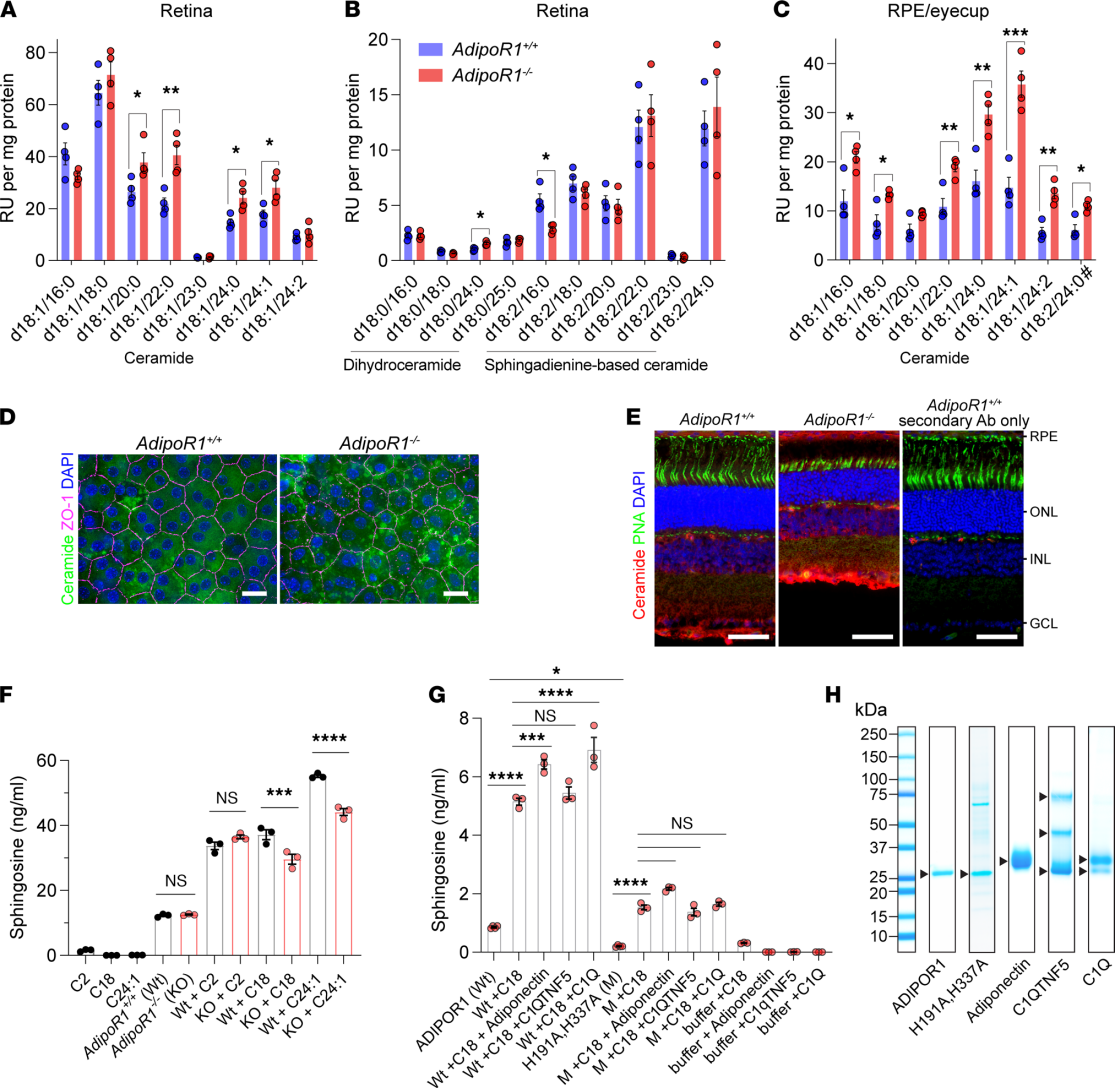
Accumulation of ceramides in the retina and RPE eyecup of *AdipoR1^–/–^* mice, and the ceramidase activity of ADIPOR1. (**A**–**C**) Ceramides were quantified by LC-MS in the retina (**A** and **B**) and RPE eyecup (**C**) of 4-week-old *AdipoR1^+/+^* and *AdipoR1*^–/–^ mice. Data represent the mean ± SEM; *n* = 4 for each genotype. The statistical significance was determined by the 2-tailed unequal variance (Welch) *t* test; *P < 0.05, **P < 0.01, ***P < 0.001. (**D**) En face images of ceramide staining (green) in RPE flatmounts of 4-week-old *AdipoR1^+/+^* and *AdipoR1^–/–^* mice. The tight junction protein ZO-1 (purple) demarcates cell boundaries. Scale bar: 20 μm. (**E**) Cross sections of the *AdipoR1^+/+^* and *AdipoR1*^–/–^ mouse retinas stained with ceramide Ab (red). Cones were stained with PNA-fluorescein (green), and cell nuclei were stained with DAPI (blue). Scale bar: 50 μm. (**F**) Enzymatic assay performed on the whole eye lysates from *AdipoR1^+/+^* and *AdipoR1*^–/–^ mice, using ceramide C2, C18, or C24:1 as substrates. (**G**) The enzymatic assay was performed with purified mouse ADIPOR1 (WT) or with the H191A,H337A-mutant (M) of ADIPOR1, using ceramide C18 as a substrate. The ceramidase activity was compared with or without adding high-molecular-weight fractions of recombinant mouse adiponectin, recombinant human C1QTNF5, or native mouse C1Q. Detected sphingosine (d18:1) values were normalized to internal standard (sphingosine-d7). The data (mean ± SEM) in **F** and **G** are representative of 3 independent experiments performed in 3 or 4 replicates. (**H**) Coomassie-stained reducing SDS-PAGE analysis of the proteins used in the assay shown in **G**. In **F** and **G**, statistical significance was determined by 1-way ANOVA followed by Sidak’s post hoc test; *P < 0.05, **P < 0.01, ***P < 0.001, ****P < 0.0001.

Liquid chromatography–mass spectrometry (LC-MS) analysis revealed that, in 4-week-old *AdipoR1*-KO mice, there was an accumulation of ceramides in both retina and RPE (Figure 5, A–C). In the retinas, there were 1.6- to 2.0-fold increases of 4 ceramides; namely, C20 (d18:1/20:0), C22 (d18:1/22:0), C24 (d18:1/24:0), and C24:1 (d18:1/24:1), as well as dihydroceramide C24 (d18:0/24:0) (Figure 5, A and B). There was also a decrease of 1 sphingadienine-based ceramide C16 (d18:2/16:0) (Figure 5B). Most of the ceramide species in the RPE eyecups were elevated, but the highest (~2.4-fold) increase was observed for ceramides C24:1 and C24:2 (d18:1/24:2) (Figure 5C). For the other ceramides C16 (d18:1/16:0), C18 (d18:1/18:0), C22, and C24, along with the sphingadienine-based ceramide C24 (d18:2/24:0), there were 1.7- to 1.8-fold increases when compared with WT. The C22, C24, and C24:1 ceramide species were elevated in both neural retina and RPE eyecup samples.

A second, independent LC-MS analysis of ceramides in 5-week-old *AdipoR1^–/–^* mice further demonstrated their accumulation in the retina and RPE eyecup. Among retinal ceramides, there was a 2.5-fold increase of C20, a 4.1-fold increase of C24:1, and 7.1- and 7.4-fold increases of C24 and C22, respectively (Supplemental Figure 2A). In the RPE eyecup, there was a ~2-fold increase of C22 and C24, and a 1.7-fold increase of sphingadienine-based ceramides C20 (d18:2/20:0) and C22 (d18:2/22:0) (Supplemental Figure 2B). In the RPE eyecup, we also noticed 2.1- to 3.1‑fold increases of some monohexosylceramides species — namely, HexC16 (d18:1/16:0), HexC18 (d18:1/18:0), HexC20 (d18:1/20:0), HexC24 (d18:1/24:0), and sphingadienine-based HexC20 (d18:2/20:0) (Supplemental Figure 2B).

To examine the localization of the accumulated ceramides, we probed the RPE eyecup, retinal flatmounts, and retinal cross-sections from 1-month-old *AdipoR1^–/–^* mice with an anti-ceramide Ab. On the retina flatmounts, the results were not conclusive, but on the RPE flatmounts, we found some organelles, plausibly phagolysosomes (23, 24), stained in an anti-ceramide Ab–specific manner (Figure 5D). There were significantly more ceramide-enriched organelles in *AdipoR1^–/–^* RPE cells, which also were larger-sized than WT RPE cells. On the retinal cross-sections, we noticed increased signals in the RPE cell body and intense Ab staining in the Bruch’s membrane side, suggesting ceramide accumulation on the RPE basal side/Bruch’s membrane in 1-month-old *AdipoR1^–/–^* mice (Figure 5E). Additionally, we observed high-intensity signals in the ganglion cell layer.

### ADIPOR1 has intrinsic ceramidase activity, which can be stimulated by adiponectin and complement component 1q (C1Q).

The lack of the *ADIPOR1* gene could result in insufficient ceramidase activity in the retinal tissues. To test this hypothesis, we prepared eye lysates from *AdipoR1^–/–^* and *AdipoR1^+/+^* mice and challenged them with ceramides C2 (d18:1/2:0), C18 (d18:1/18:0), or C24:1 (d18:1/24:1) (Figure 5F). We detected ceramidase activity in eye lysates derived from *AdipoR1^+/+^* and *AdipoR1^–/–^* mice, although the latter generated 19.7% and 21.6% less sphingosine from C18 and C24:1, respectively, which indicates decreased ceramidase activity in the eyes of the *AdipoR1^–/–^* mice. There was no difference in sphingosine production when using C2 as a substrate, implying that ADIPOR1 hydrolyzes ceramides with longer 18- and 24-carbon fatty acyl chains rather than the short ones.

Next, we specifically wanted to confirm that ADIPOR1 possesses ceramidase activity, using pure recombinant protein expressed in mammalian cells. Furthermore, we investigated if it is possible to eliminate the activity by mutating the conserved histidine residues (H191, H337, H341) that coordinate zinc in the putative catalytic site (11). We designed a mutant containing 2 alanine residues substituted for His residues, namely H191A and H337A, since Holland and colleagues have previously shown that mutating H191 alone was not sufficient to terminate the activity — only to reduce it by roughly 33% (1). We produced in mammalian cells and isolated both proteins, WT ADIPOR1 and the H191A,H337A-mutant. Due to the quickly decaying enzymatic activity of purified receptors, we isolated both proteins within ~12 hours and set up enzymatic assays immediately after purification to retain the maximum ceramidase activity. Additionally, we noticed that FLAG peptides present in the preparations after elution had a positive effect on preserving the activity, possibly due to a stabilizing effect on the receptors. We observed potent ceramidase activity of the WT ADIPOR1, which hydrolyzed both C18 and C24:1, although hydrolysis of C24:1 was less effective (Figure 5G and Supplemental Figure 9). We also found that the H191A,H337A-mutant had 3.4-fold and 2.3-fold lower activity than WT ADIPOR1 when tested with C18 and C24:1, respectively (Figure 5G and Supplemental Figure 9). However, the remaining activity might also come from other copurified ceramidases plausibly present in the preparation of the H191A,H337A-mutant, which contained some residual impurities in contrast to the highly pure WT ADIPOR1 (Figure 5H).

Moreover, since adiponectin was shown to stimulate the ADIPOR1/2 ceramidase activity, we further investigated if other proteins containing a trimeric C1q globular domain analogous to adiponectin could exert similar effects (1, 11). Therefore, in addition to adiponectin, we investigated the effect of high-molecular-weight (HMW) fractions of C1QTNF5 and C1Q on ADIPOR1 ceramidase activity. Our results demonstrated that adiponectin and C1Q increased the ADIPOR1 activity toward C18 hydrolysis by 24.7% and 34.4%, respectively, while C1QTNF5 did not show significant stimulation (Figure 5G). When C24:1 was used as a substrate, adiponectin increased activity by 21.8% and C1Q by 23.9% (Supplemental Figure 9). None of these C1Q proteins significantly enhanced the activity of the H191A,H337A-mutant toward C18 or C24:1, suggesting that histidine mutations impair not only the intrinsic ceramidase activity, but plausibly also affect the ADIPOR1 capacity for being stimulated by agonists. Interestingly, ADIPOR1 had 3.5-fold more sphingosine bound than did the H191A,H337A-mutant, indicating that the mutant has a lower capacity for binding/retaining sphingosine.

### AdipoR1 expression profile in the mouse and human retina.

To examine the expression pattern of *ADIPOR1*, *ADIPOR2,* and other genes involved in ceramide metabolism in different retinal cell types and the RPE, we performed single-cell RNA-Seq (scRNA-Seq) analysis of ocular cells originating from humans and mice. To visualize clusters corresponding to distinct cell types, the data were analyzed with the t-distributed stochastic neighbor embedding (t-SNE) algorithm (Supplemental Figure 4, A and C). For both humans and mice, *ADIPOR1* was expressed in all retinal cell types, and as expected, its expression was markedly higher than that of *ADIPOR2*, which showed minimal expression levels in both species except for endothelial cells in humans (Supplemental Figure 4, B and D). In human cells, expression levels of *ADIPOR1* appeared in the following order: highest in the cones, followed by rods, and then relatively lower in all other cell types, including RPE (Supplemental Figure 4B). The highest *ADIPOR1* expression in mouse cells was observed in rods, followed by RPE cells. *ADIPOR1* was the most highly expressed ceramide hydrolase in mice and one of the 2 most highly expressed ceramidases in humans, next to acid ceramidase *ASAH1* (Supplemental Figure 4, B and D).

### RNA-Seq analysis of the retina and RPE from AdipoR1^–/–^ mice.

To investigate how the lack of *AdipoR1* affects gene expression profiles, we performed a bulk RNA-Seq analysis of the retina and RPE from *AdipoR1^–/–^* and WT mice at P30. In the retina, we found 1582 differentially expressed genes (DEGs; fold change ≥ 1.5, FDR < 5%), of which 1292 (81.7%) were upregulated and 290 (18.3%) were downregulated. In the RPE, we found 1746 DEGs, of which 712 (40.8%) were upregulated and 1034 (59.2%) were downregulated. Over 10% more DEGs were found in the RPE than in the retina, and ~3.6-fold more DEGs were downregulated in the RPE, while ~1.5-fold more DEGs were upregulated in the retina. Overall, these results indicate that gene expression patterns were more affected in the RPE compared with the neural retina of *AdipoR1^–/–^* mice.

**Figure 6:**
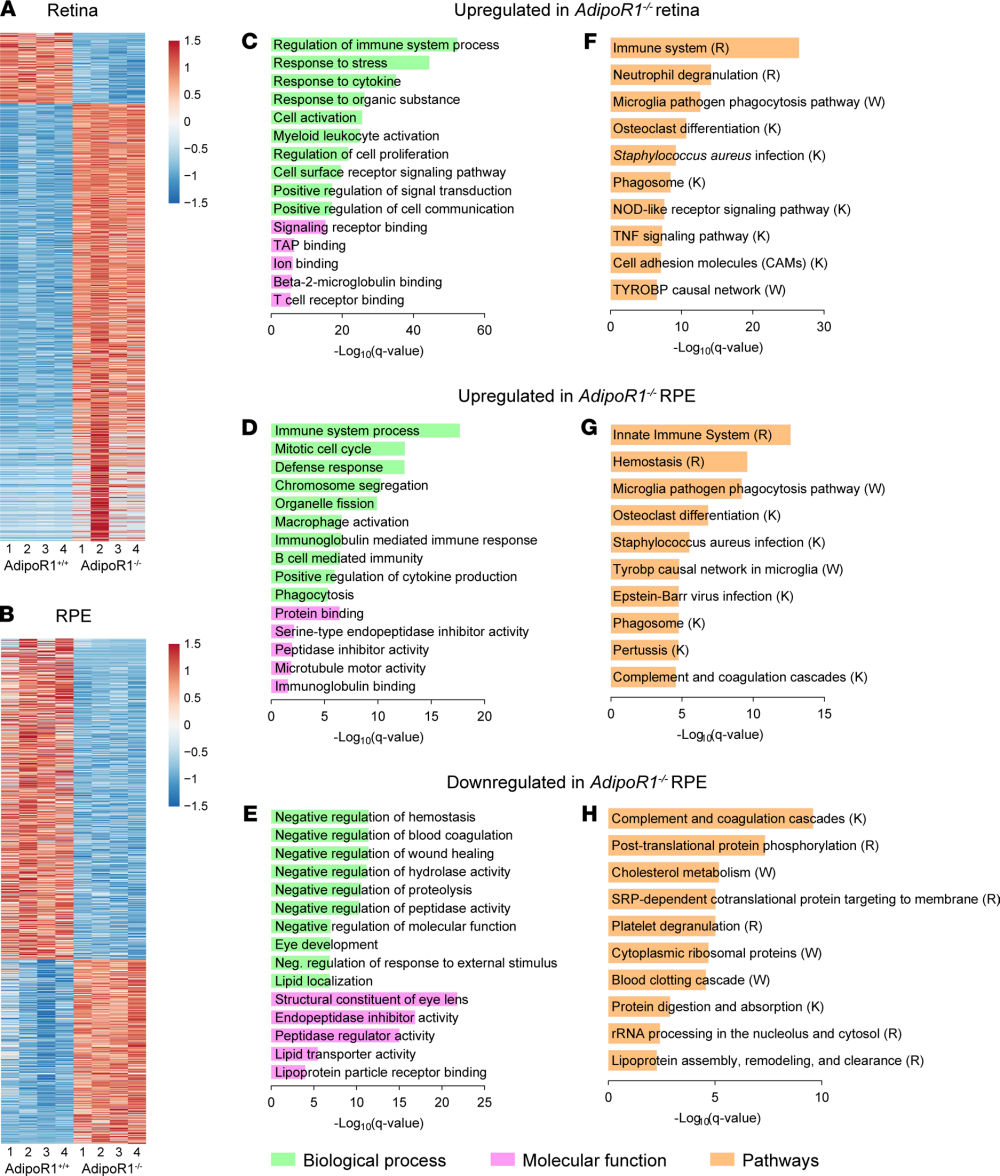
Bulk RNA-Seq analysis of the retina and RPE from *AdipoR1^–/–^*. (**A** and **B**) Heatmaps of population RNA-Seq data, showing all the differentially expressed genes (DEGs) for *AdipoR1^–/–^* versus WT mice in the retina (**A**) and RPE (**B**). Color bars show the relative gene expression values (*n* = 4 mice, at P30). (**C**–**E**) Top GO terms (biological process and molecular function) linked to DEGs in the retina and RPE of *AdipoR1^–/–^* mice. (**F**–**H**) Top pathways linked to DEGs in the retina and RPE of *AdipoR1^–/–^* mice. R, Reactome; K, KEGG; W, Wikipathways.

A hierarchical clustering heatmap was generated to represent the up- and downregulated genes in the retina and RPE (Figure 6, A and B). Subsequently, all DEGs were used in gene ontology (GO) and pathway analyses. The upregulated DEGs in *AdipoR1^–/–^* mice were involved in various biological processes, most of which were common for both retina and RPE, such as immune system processes, response to stress and cytokines, and activation of leukocytes (Figure 6, C–E), and pathways related to microglia activation and phagocytosis were also shared among retina and RPE (Figure 6, F–H).

Gene enrichment analysis in RPE also showed upregulation of genes involved in organelle fission, as well as microtubule motor activity (Figure 6D). Both events could be related to dysfunctional microtubules and might be linked to the enlarged organelles in the RPE of *AdipoR1^–/–^* mice (shown in Figure 5D) and the subsequent compensatory mechanisms leading to increased expression of genes engaged in organelle fission. Downregulated DEGs in the RPE were associated with diverse processes related to tissue repair and were also involved in eye and lens development, lipid localization and transport, lipoprotein binding, and cholesterol metabolism. Furthermore, we found significant downregulation of genes involved in (a) posttranslational protein phosphorylation; and (b) protein digestion and absorption — the latter suggesting impaired phagocytosis in RPE cells.

**Figure 7:**
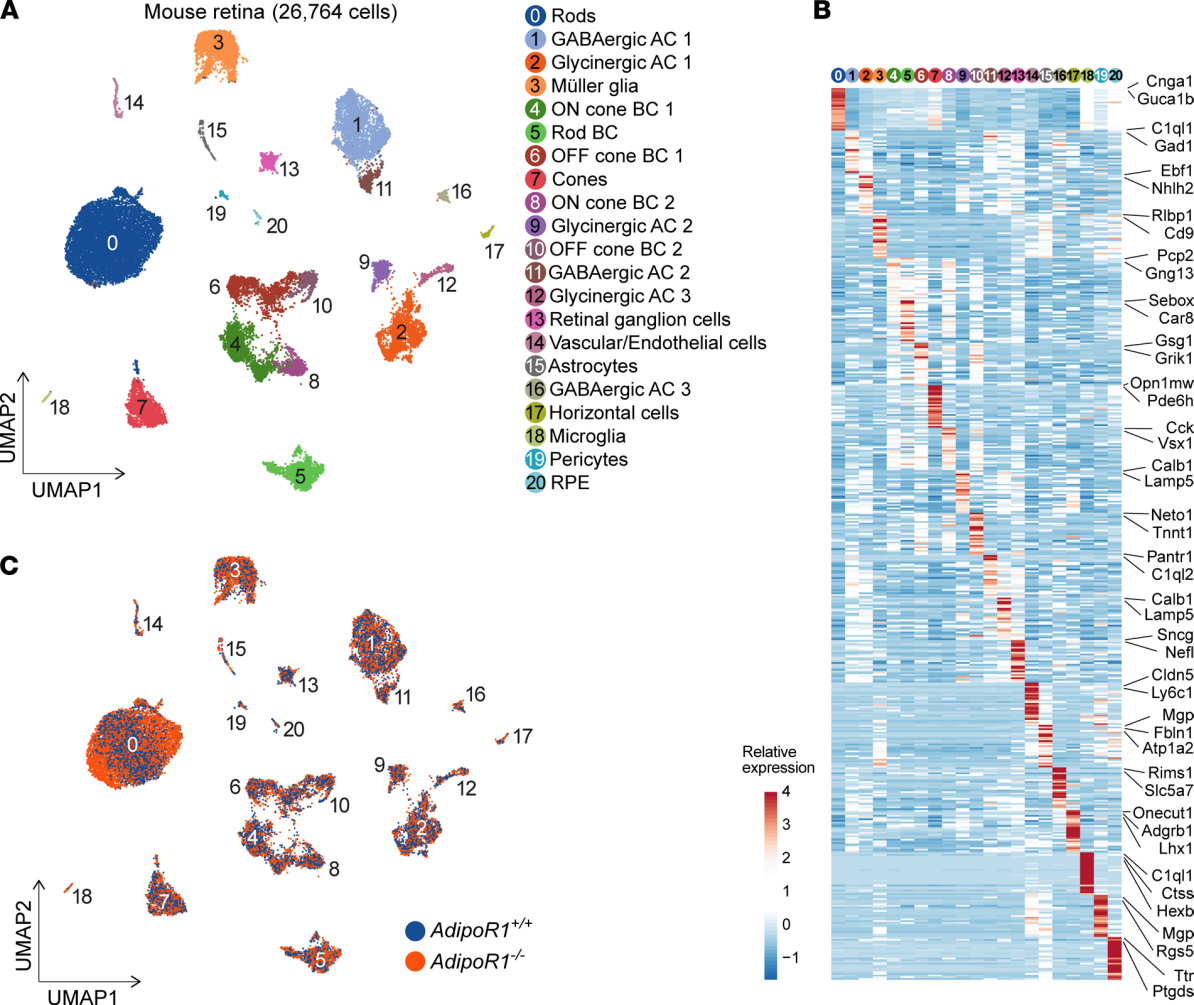
Single-cell RNA-Seq analysis of *AdipoR1^–/–^* and WT retinas at P19, P25, and P30. (**A**) Clustering of retinal cell types obtained from both *AdipoR1^–/–^* and *AdipoR1^+/+^* mice at P19, P25, and P30 (*n* = 12; 2 mice for each time point and genotype). (**B**) Characterization of clusters: cell annotation and DEGs specific to clusters (shown on the right). The color bar indicates the relative expression values. (**C**) Clustered cells were color-coded according to genotype: *AdipoR1^+/+^* labeled blue and *AdipoR1^–/–^* colored orange. GABAergic, γ-aminobutyric acid–releasing; AC, amacrine cells; BC, bipolar cells.

**Figure 8:**
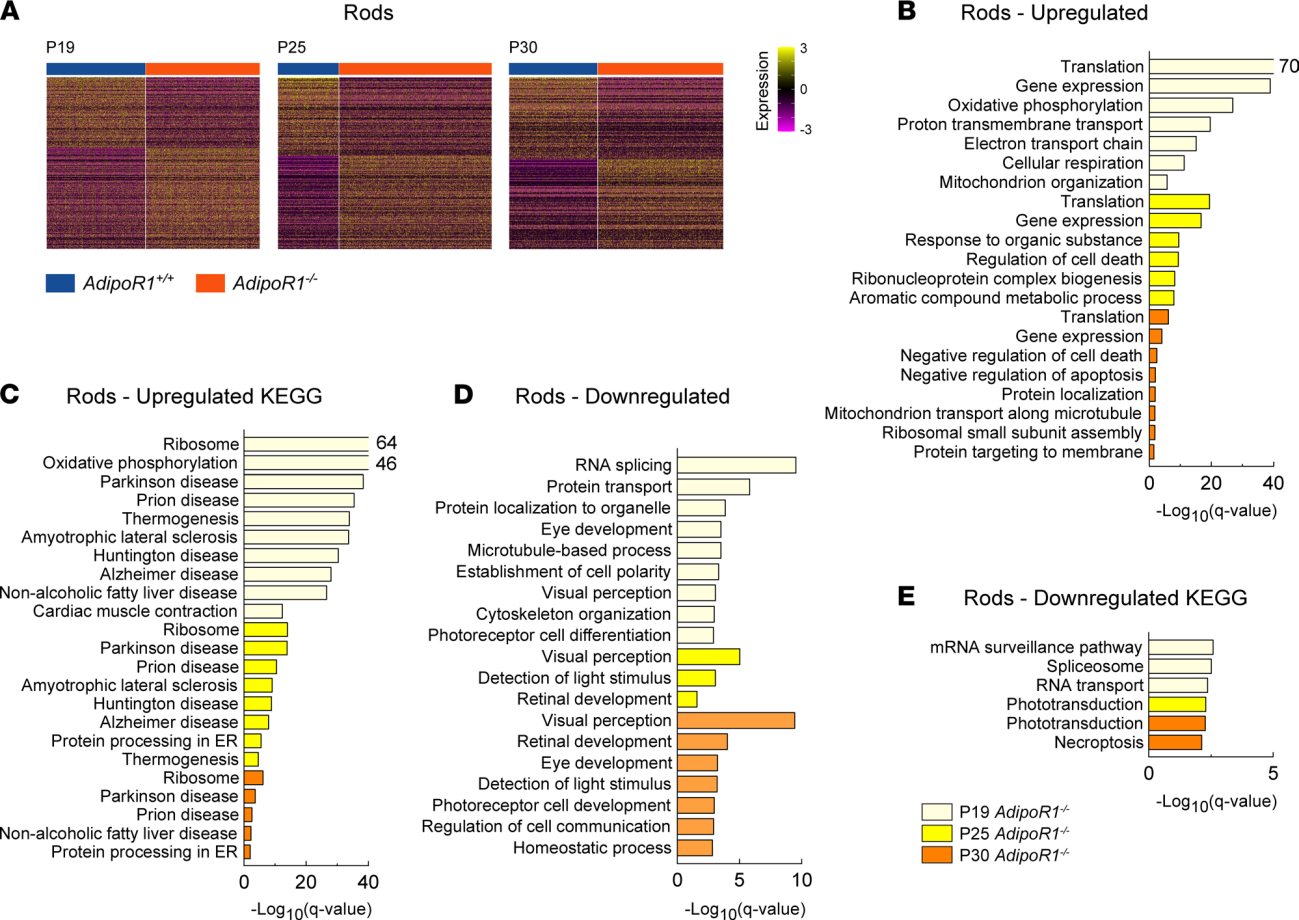
Transcriptome analysis of rod photoreceptors in *AdipoR1^–/–^* and WT mice at P19, P25, and P30. (**A**) DEGs in rods of *AdipoR1^+/+^* (blue) and *AdipoR1*^–/–^ (orange) mice at P19, P25, and P30 (*n* = 12; 2 mice for each time point and genotype). (**B** and **D**) The most enriched GO terms (biological processes). (**C** and **E**) Top KEGG pathways found in rods based on DEGs shown in **A**.

To better understand the altered gene expression patterns in rod photoreceptors, the most affected cell type in *AdipoR1^–/–^* retina, we performed scRNA-Seq on the retina from *AdipoR1^–/–^* and WT mice at different ages. We chose animals at P19, P25, and P30 to learn what events occur before the onset, after the onset, and at the advanced stage of photoreceptor degeneration, respectively. We profiled 26,764 cells from the retina and RPE of the KO and WT mice at different ages; these profiles were combined and subjected to removal of genotype differences between the KO and WT mice to enable clustering based solely on the cell type–specific factors. Subsequently, we segregated cells into 21 unique clusters (Figure 7A), identifying all major retinal cell types indicated by cluster annotation (Supplemental Figure 8, A and B). Then, we characterized cell clusters and identified cell type–specific genes, from which the top 2–3 genes created unique gene signatures (Figure 7B). We color-coded cell clusters according to the genotype, which emphasized substantial differences in rod photoreceptors and most other cell types in the *AdipoR1^–/–^* mice compared with WT (Figure 7C). We analyzed DEGs in each cell type, focusing on rod photoreceptors (Figure 8A). Finally, we assessed biological processes and pathways enriched in rods by the identified DEGs at 3 different stages of retinal degeneration in *AdipoR1^–/–^* mice (Figure 8, B–E). GO analysis showed that, before the onset of retinal degeneration (at P19), there was upregulation of genes involved in translation, oxidative phosphorylation, and mitochondrion organization (Figure 8B), which was also reflected in the upregulated KEGG pathways (Figure 8C). The upregulated translation is the top event common to all 3 examined ages. Also, among the upregulated KEGG pathways, many are related to neurodegenerative diseases, such as Parkinson’s, Huntington’s, Alzheimer’s, prion disease, or amyotrophic lateral sclerosis. Repressed processes are mostly RNA splicing and protein localization, but also include processes related to microtubules, cytoskeleton organization, photoreceptor differentiation, and visual perception — the last being the top downregulated pathway at the P25 and P30 ages (Figure 8, D and E).

### Inhibition of ceramide generation increases photoreceptor survival in AdipoR1^–/–^ mice.

**Figure 9:**
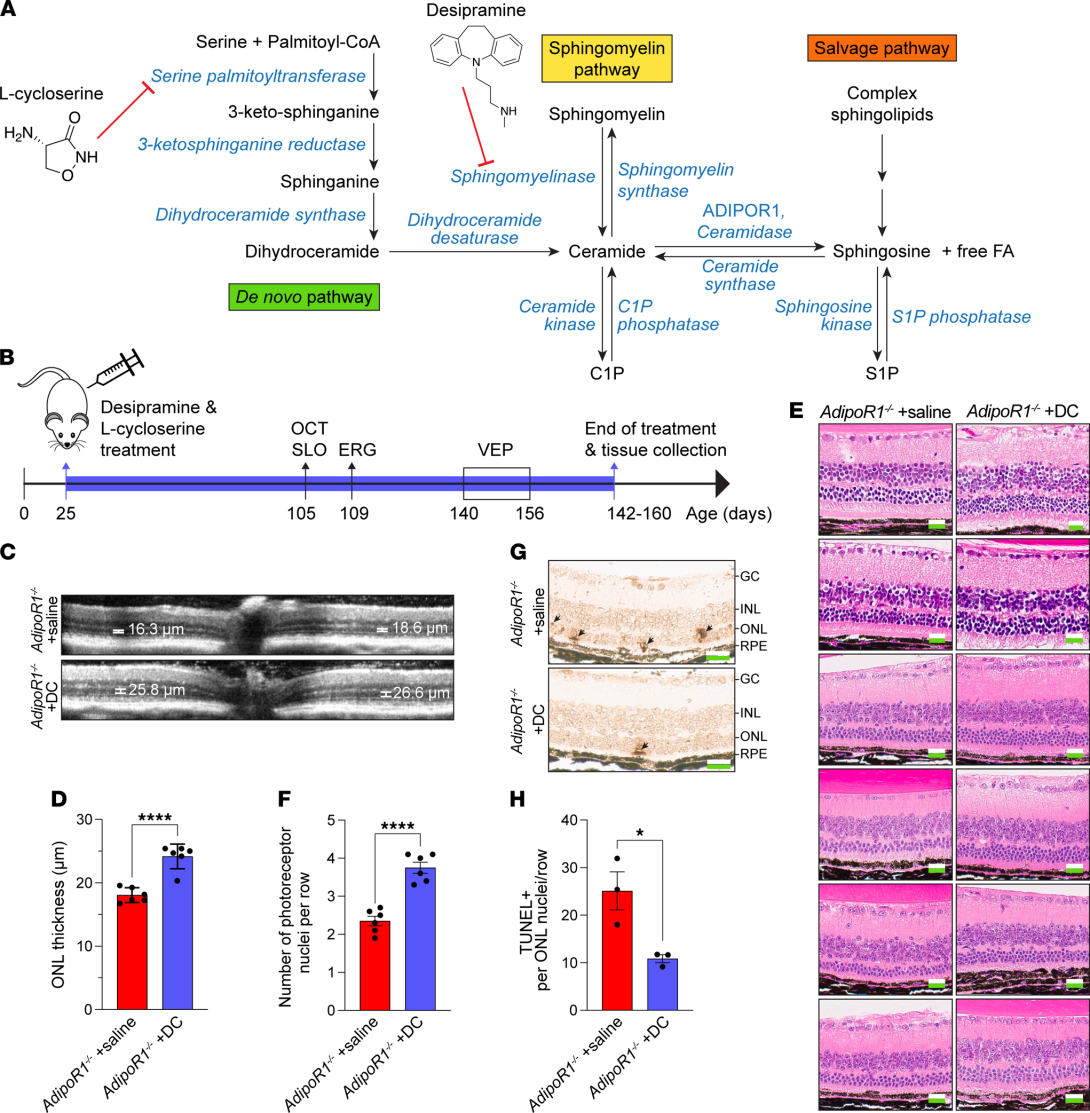
I.p. injection of desipramine/L-cycloserine (DC) cocktail increased photoreceptor survival in *AdipoR1^–/–^* mice. (**A**) Depictions of pharmacological inhibition of the ceramide de novo synthesis (left) and sphingomyelinase pathways (right) with L-cycloserine and desipramine, respectively. (**B**) Experimental protocol outline: 2 groups of *AdipoR1^–/–^* mice (*n* = 6) were treated with saline or DC 3 times a week, starting from P25 to P142–P160 (~17–19 weeks). At P105, OCT and SLO measurements were done; at P109, ERG measurements were done; and at P140–P156, VEP measurements were performed. After VEP analysis, the mice were euthanized, and their eyes were collected for histology and MS analyses. (**C**) Representative OCT images are shown of retinas from saline-treated and DC-treated *AdipoR1*^–/–^ mice at P105 (after ~11-week treatment). White calipers at the inferior and superior aspects of the section indicate the ONL thickness measured 500 μm from the ONH. (**D**) The average ONL thickness of both eyes is shown (*n* = 6). (**E**) H&E-stained sections of retinas from saline- or DC-treated *AdipoR1*^–/–^ mice, demonstrating a higher number of photoreceptor nuclei per row in the DC-treated group. (**F**) Data from **E** quantified. Images were taken 500–750 μm from the ONH (*n* = 6). Scale bar: 20 μm. (**G**) TUNEL staining of the retinal histologic section, showing a decreased number of TUNEL^+^, apoptotic photoreceptor nuclei in the DC- versus saline-treated *AdipoR1*^–/–^ mice (dark brown nuclei, marked by arrows). Scale bar: 20 μm. (**H**) Quantification of the TUNEL^+^ nuclei averaged from 3 sections per mouse (*n* = 3). In **D**, **F**, and **H**, data represent the mean ± SEM, and the statistical significance was determined by the 2-tailed Student’s *t* test; *P < 0.05, ****P < 0.0001.

To examine whether we could improve photoreceptor survival in *AdipoR1^–/–^* mice by inhibiting 2 of the 3 ceramide-generation pathways, de novo biosynthesis, and sphingomyelin hydrolysis, we pharmacologically treated the mice and assessed the treatment outcome (Figure 9A). We decided to use a combination of 2 drugs, desipramine/L-cycloserine (DC), which were previously shown to effectively lower ceramide levels (25–30). To test if lowering ceramide levels would have a protective effect on photoreceptor cells, 2 groups of *AdipoR1^–/–^* mice (*n* = 6) were administered either saline or DC by i.p. injection (3 times per week), starting from P25 to P142–P160 (for ~17–19 weeks) (Figure 9B); the mice were examined by OCT, SLO, ERG, and visually evoked potential (VEP) measurements during the treatment. Subsequently, the mice were euthanized, and their eyes were collected for histology, TUNEL assay, and ceramide quantification by LC-MS.

After 80 days of DC treatment, we assessed retinal morphology with OCT and SLO. The OCT showed a significant 34% increase in ONL thickness in the DC- versus saline-treated *AdipoR1^–/–^* mice (Figure 9, C and D, and Supplemental Figure 5A), suggesting that DC partially protected photoreceptor cells from dying. On the SLO scans, there were no significant differences (Supplemental Figure 5B).

To further assess the morphological effects of prolonged (~17–19 weeks) treatment with DC, we counted the number of photoreceptor nuclei per row in the ONL at P142–P160 (~20–23 weeks old). The number of photoreceptors in the *AdipoR1^–/–^* mice was more than 37% higher in the DC-treated group, confirming a protective effect of DC on photoreceptor survival (Figure 9, E and F). Photoreceptor survival was also assessed with TUNEL staining for apoptosis (Figure 9G). The results demonstrated a reduction in photoreceptor cell death, as indicated by a significant decrease in TUNEL^+^, apoptotic cells in the DC- versus saline-treated *AdipoR1^–/–^* mice (Figure 9H).

**Figure 10:**
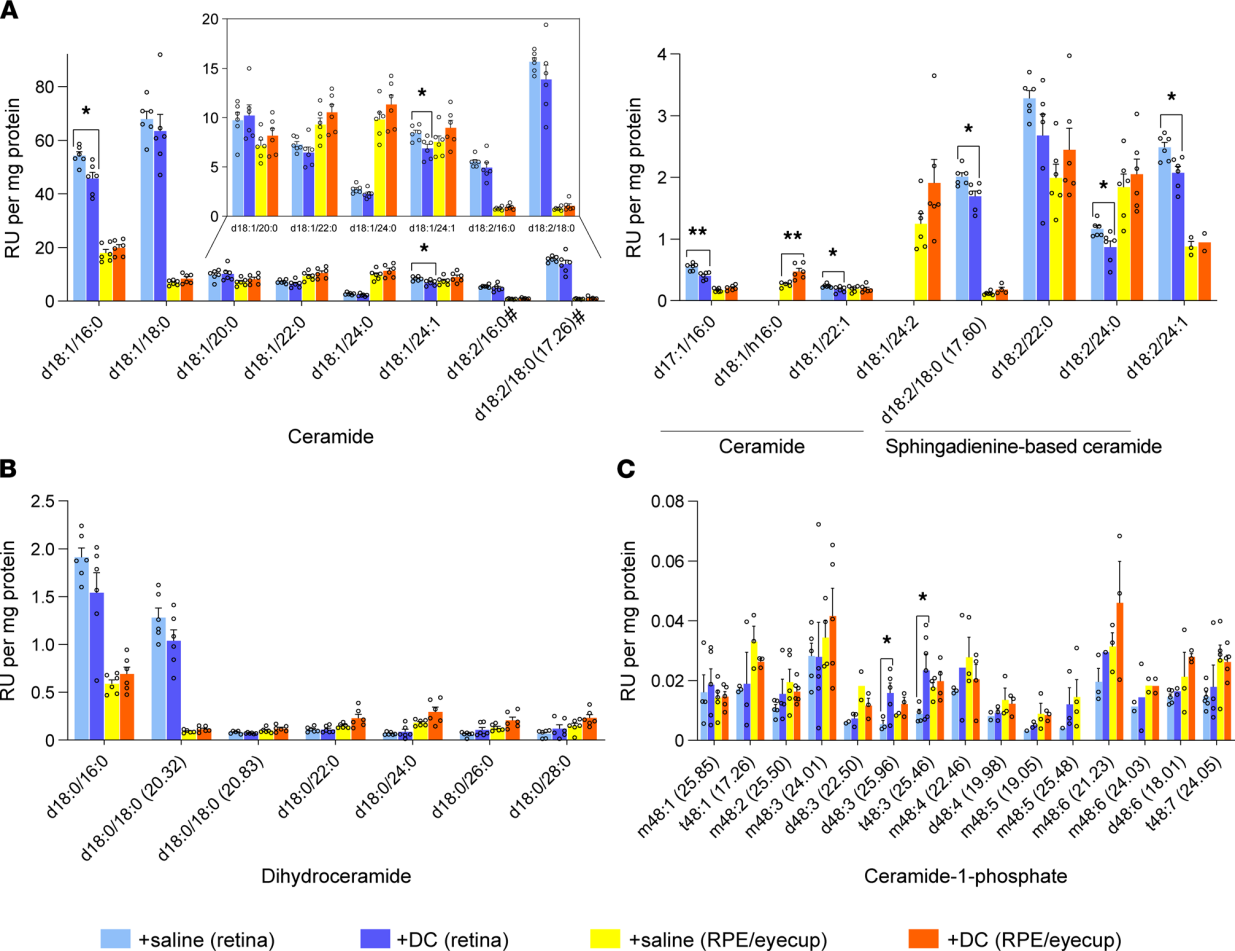
Desipramine/L-cycloserine (DC) treatment lowers ceramide levels in the retinas of *AdipoR1^–/–^* mice. Two groups of *AdipoR1*^–/–^ mice were i.p.-injected with either saline or DC 3 times a week for ~17–19 weeks. After treatment, ceramides in the neural retinas and RPE eyecups were quantified by LC-MS. (**A**–**C**) Ceramides and sphingadienine-based ceramides (on the left chart marked with #) (**A**), dihydroceramides (**B**), and ceramide-1-phosphate species (**C**) are shown. Numbers in parentheses indicate the retention times in minutes. Data represent mean ± SEM; *n* = 6 for both groups. Statistical significance was determined by the 2-tailed unequal variance (Welch) *t* test; *P < 0.05, **P < 0.01.

We also examined the effects of long-term DC treatment on ceramide and sphingosine levels. In retinas of *AdipoR1^–/–^* mice, we observed decreases of 7 ceramide species, including ceramides C16 (d18:1/16:0), C22:1 (d18:1/22:1), and C24:1 (d18:1/24:1) and heptadecasphingosine-based ceramide C16 (d17:1/16:0) by 16%, 24%, 18%, and 27%, respectively; and sphingadienine-based ceramides C18 (d18:2/18:0), C24 (d18:2/24:0), and C24:1 (d18:2/24:1) by 16%, 24%, and 17%, respectively (Figure 10A). We did not see significant changes in dihydroceramide levels in DC-treated versus saline-treated *AdipoR1^–/–^* mice (Figure 10B). Moreover, in the retinas of DC-treated *AdipoR1*-KO mice, there were increases of 2 ceramide-1-phosphate species d48:3 and t48:3 by 190% and 163%, respectively (Figure 10C). Conversely, in the RPE eyecups, there was an increase in ceramide d18:1/h16:0 by 79%, and there were no changes in dihydroceramide or ceramide-1-phosphate levels in the DC-treated compared with saline-treated *AdipoR1^–/–^*mice (Figure 10A). Since free sphingosine appears to derive solely from the breakdown of ceramide and complex sphingolipids (31), we expected that lowering ceramide generation through inhibition of de novo synthesis and sphingomyelin hydrolysis would also decrease sphingosine levels in DC-treated *AdipoR1^–/–^* mice. However, we did not observe significant changes in sphingosine, sphinganine, sphingosine-1-phosphate, or sphinganine-1-phosphate (Supplemental Figure 6).

### Lowering ceramide levels improves retinal function and restores near-normal responses in the primary visual cortex of the AdipoR1^–/–^ mice.

**Figure 11:**
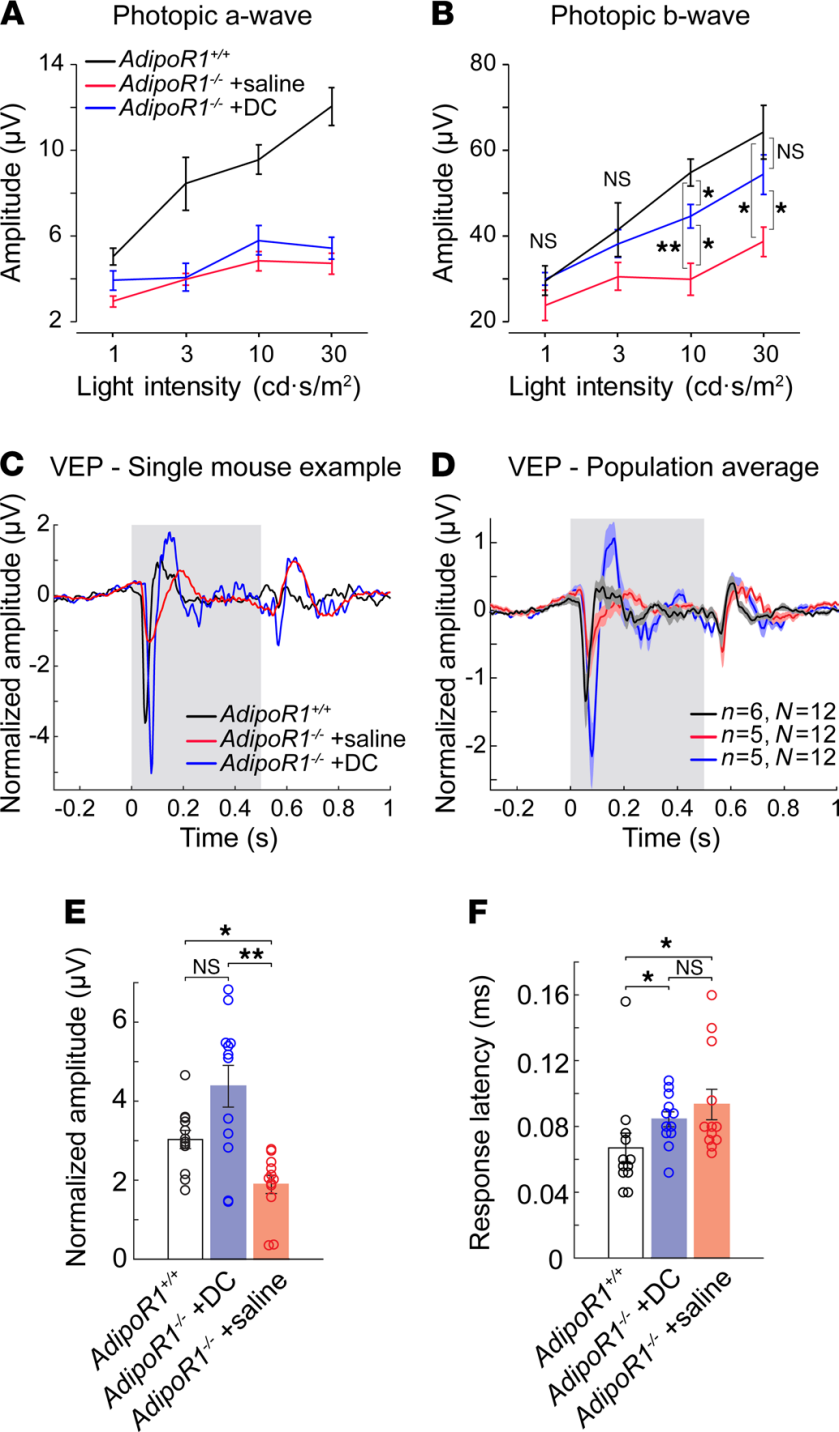
Desipramine/L-cycloserine (DC) treatment restores balance to the visual system in *AdipoR1*^–/–^ mice. (**A** and **B**) ERG recordings of photopic a-wave amplitudes (**A**) and b-wave amplitudes (**B**) in *AdipoR1^+/+^* or *AdipoR1*^–/–^ mice treated with saline or DC for 12 weeks (*n* = 6 for all groups). DC or saline was administered 3 times a week by i.p. injection. (**C**) Examples of the flash evoked potentials (VEP) from a single *AdipoR1^+/+^* mouse (black), DC-treated *AdipoR1*^–/–^ mouse (blue), and saline-treated *AdipoR1*^–/–^ mouse (red). (**D**) Population average for each group. *n* indicates the number of animals used, and *N* indicates the number of recording sites used for statistics. (**E**) Comparison of the average VEP amplitudes. (**F**) Comparison of the response latencies for each animal group. All data are mean with SEM. In **A** and **B**, statistical significance was determined with repeated measures 2-way ANOVA, followed by Sidak’s post hoc correction. In **E** and **F**, Kruskall-Wallis analysis followed by Dunn-Sidak multiple comparison correction was used; *P < 0.05, **P < 0.01.

To determine whether the protective effect of DC treatment on photoreceptor cells was associated with maintenance of retinal function, we recorded ERG responses under light-adapted (cone-mediated) conditions in *AdipoR1^–/–^* mice treated with DC or saline, as well as in age-matched WT animals (Figure 11, A and B). In photopic ERGs, we did not observe any differences in the a-wave amplitudes (Figure 11A). In contrast, the amplitude of the photopic b-wave, which is proportional to the overall response of bipolar cells and Müller glia, improved substantially at higher light intensities (10 and 30 cd·s/m^2^) in the DC-treated versus saline-treated *AdipoR1^–/–^* mice, to the extent that the response was not significantly different from WT mice at 30 cd·s/m^2^ (Figure 11B). In rodent ERGs, the amplitude of the b-wave is a generally accepted indicator of the quality of visual function (32).

Lastly, we assessed if prolonged DC treatment could restore the integrity of visual image transmission to the visual cortex and cortical processing of the visual information. We tested visually evoked responses in 3 groups of mice: untreated WT control (*AdipoR1^+/+^*, *n* = 6), DC-treated KO mice (*n* = 5; *AdipoR1^–/–^* + DC), and saline-treated KO mice (*n* = 5; *AdipoR1^–/–^* + saline). The first stage of our vision assessment was based on comparing amplitudes of VEPs in response to a brief flash of light. Representative examples from single mice (Figure 11C) and population average VEPs (Figure 11D) revealed big differences between the studied groups. The *AdipoR1^–/–^* animals treated with the DC drug cocktail had a greater average VEP amplitude (4.4 ± 0.53 μV, ± SEM) than saline-treated *AdipoR1^–/–^* mice (1.9 ± 0.23 μV, ± SEM), and WT (3 ± 0.23 μV, ± SEM); however, the latter was not statistically significant (Figure 11E). Whereas the firing rate of response was greatest in DC-treated animals, the response latency was longer for both DC- and saline-treated *AdipoR1*-KO animals than for WT controls (Figure 11F).

To investigate potential vision improvements in more detail, we compared peak responses of single cells recorded from the primary visual cortex for the 3 experimental groups of animals. Representative single-cell examples show increased activity in both DC-treated (Supplemental Figure 7B) and saline-treated (Supplemental Figure 7C) *AdipoR1^–/–^* mice compared with the WT controls (Supplemental Figure 7A). However, only the increase in response between the DC-treated and control groups was statistically significant (Supplemental Figure 7D). In addition, both DC- and saline-treated *AdipoR1^–/–^* groups showed elevated background activity compared with controls (Supplemental Figure 7E).

**Figure 12:**
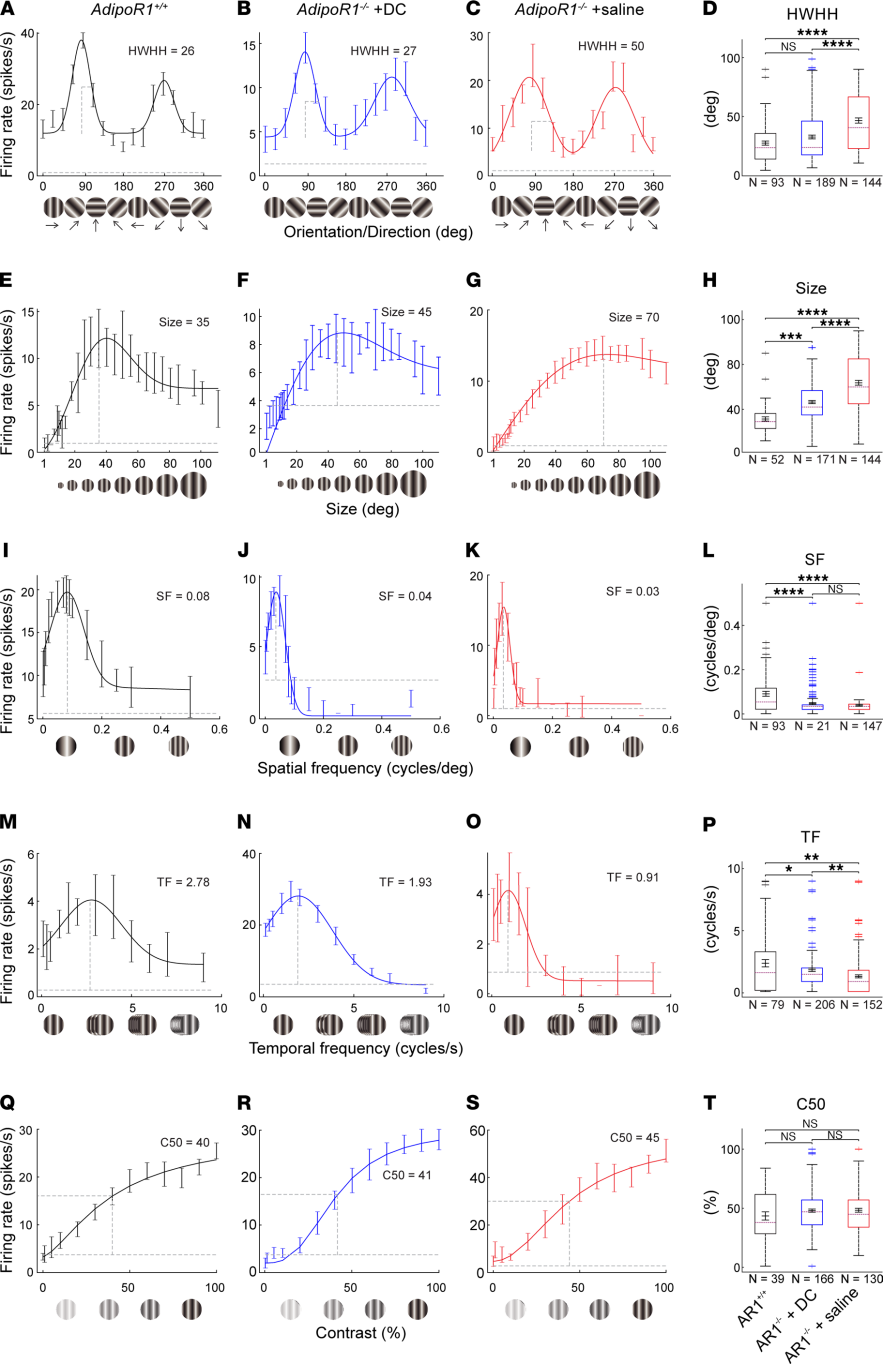
Comparison of V1 neuron tuning curves in response to drifting gratings. (**A**–**C**) Single neuron orientation tuning curves are shown from control *AdipoR1^+/+^* (**A**), DC-treated *AdipoR1^–/–^* (**B**), and saline-treated *AdipoR1^–/–^* mice (**C**). (**D**) Comparison of orientation tuning width (HWHH) across the 3 groups. (**E**–**T**) Representative tuning curves are shown for each group and comparisons of population averages for stimulus size (**E**–**H**), spatial frequency (SF) (**I**–**L**), temporal frequency (TF) (**M**–**P**), and contrast (**Q**–**T**). The horizontal dashed lines indicate background activity; the vertical dashed lines indicate optimal parameters. For all panels, *n* = 6 for *AdipoR1^+/+^*, and *n* = 5 for both *AdipoR1^–/–^* groups. *N* indicates the number of cells. Boxes extend from the 25th to the 75th percentiles. The dashed purple line indicates the median. Error bars inside the boxes indicate mean ± SEM. The whiskers extend to the most extreme data points that are not considered outliers; crosses beyond whiskers indicate outlying values. Statistical significance was determined with Kruskall-Wallis analysis followed by Dunn-Sidak multiple comparison correction; *P < 0.05, **P < 0.01, ***P < 0.001, ****P < 0.001.

To obtain more detailed information about the visual physiology of the 3 animal groups, we compared tuning curves and optimal parameters of V1 cortex neuron responses to drifting-grating stimuli. Representative tuning curves for single cells are shown in Figure 12. The population data indicate significantly higher selectivity in DC-treated *AdipoR1^–/–^* mice compared with saline-treated *AdipoR1^–/–^* mice for orientation (Figure 12D), size (Figure 12H), and temporal frequency (Figure 12P), as reflected in the tuning curve examples. In particular, orientation tuning width (HWHH) was improved in DC-treated *AdipoR1^–/–^* mice to the extent that it was indistinguishable from that for *AdipoR1^+/+^* mice (Figure 12D). This correspondence was also seen in the 2 examples where the tuning width of the preferred direction for the V1 neuron response was 26° for WT (Figure 12A) and 27° for DC-treated mice (Figure 12B). For spatial frequency (Figure 12L) and contrast response (Figure 12T), there were no significant differences between DC- and saline-treated *AdipoR1^–/–^* mouse groups.

## Discussion

In this study, we characterized the effects of *AdipoR1* KO on mouse retinal structure and function. The absence of *AdipoR1* led to progressive photoreceptor degeneration resulting in a 66% decrease in the ONL thickness and in only 4 loosely organized nuclei layers remaining at 4 months of age. Furthermore, we observed a massive reduction of ERG amplitudes, particularly in scotopic conditions, suggesting primary dysfunction in the rod pathway. Additionally, there was reduced cone opsin expression and degeneration of cone outer segments even at a young age (1 month old). The retinoid visual cycle is severely impaired in *AdipoR1^–/–^* mice for all age groups, and this manifests in a dramatic drop of retinoid content in the retina.

### Localization of the ADIPOR1 protein and its expression patterns in the retina.

We found that ADIPOR1 protein localizes predominantly at the interface between the RPE apical processes and the distal part of the photoreceptor outer segments. However, the ADIPOR1 protein content in RPE cells was nearly 28-fold higher than in the neural retina. This finding is partially consistent with the previous report that used the same Ab, showing that ADIPOR1 protein localizes at the interface of photoreceptor outer segments and the RPE (5). Nevertheless, the authors concluded that ADIPOR1 is enriched in the OS rather than the RPE or OS/RPE. Here, our Western blot quantification indicated a higher ADIPOR1 content in the RPE than in the neural retina.

scRNA-Seq analysis of mouse retina indicated the highest *AdipoR1* expression in rods, followed by RPE, cones, astrocytes/endothelial cells, and microglia. This result differs from the immunodetection of ADIPOR1 protein localization discussed above. One of the possible explanations could be a higher turnover of the ADIPOR1 protein in the OS due to the fast dynamics of photoreceptor OS shedding compared with a relatively stable pool of ADIPOR1 in the RPE. Besides, it has been shown that the same protein can have different lifetimes depending not only on the cell type, but also on the subcellular localization of the protein, potentially reflecting a differential regulation of protein degradation in these compartments (33). This differential degradation is supported by many reports that have found a discrepancy between the RNA level and the actual protein abundance, depending on the tissue or cell type (5, 34, 35).

### Ablation of AdipoR1 leads to ceramide accumulation and photoreceptor death.

LC-MS analysis of ceramides in 4-week-old *AdipoR1^–/–^* mice indicated their accumulation in the RPE eyecup and in the retina. In 5-week-old KO mice, the accumulation was even more evident, especially in the retina. The ceramide build-up was further confirmed by anti-ceramide Ab staining of retinal cross-sections and RPE flatmounts, which indicated ceramide-rich organelles, plausibly phagolysosomes (24), that were bigger and more abundant in the *AdipoR1^–/–^* RPE compared with WT. Similarly, Lin and colleagues showed that ceramide accumulation in lysosomes leads to a robust increase in the number and size of lysosomes (36). Intriguingly, our RNA-Seq analysis in the RPE of *AdipoR1^–/–^* mice also showed upregulation of genes related to organelle fission, possibly suggesting a compensatory mechanism toward the defective fission processes. Moreover, others have shown that excess ceramides decrease membrane liquidity and curvature (37), destabilizing vesicles and affecting endocytosis (38). Additionally, as suggested by our RNA-Seq data from RPE cells, these processes could be further disturbed by ceramide-driven acetylation and stiffening of microtubules, which impair the organelle traffic (30, 39).

Furthermore, we found microglia/macrophage cells in the subretinal space before the onset of photoreceptor degeneration in *AdipoR1^–/–^* mice at P20. Then, in 7-week-old KO mice, we observed massive infiltration of microglia/macrophage cells to the RPE-photoreceptor interface and breakdown of the RPE barrier. Activation of the microglia/macrophage cells could suggest ongoing photoreceptor cell death (40, 41). We interpret that this process is activated or enhanced by the accumulated ceramides (15, 16, 29, 42–45).

### Altered gene expression in rods of AdipoR1^–/–^ mice and its association with neurodegenerative diseases.

Gene enrichment analysis in rods showed that, before the onset of photoreceptor degeneration (at P19), there is upregulation of genes involved in translation, oxidative phosphorylation, and mitochondrion organization — the first being the top event at all examined ages. Repressed processes are mostly RNA splicing and protein localization, which — combined with increased translation — can plausibly result in the production of erroneous/misfolded protein aggregates accumulating in the cell. This pathological event is the hallmark of neurodegenerative diseases, such as Parkinson’s, Alzheimer’s, or prion disease (46, 47), which are also overrepresented as the top pathways enriched by the upregulated DEGs in rods of *AdipoR1^–/–^* mice.

Interestingly, ceramides are accumulated in postmortem brains and altered in the plasma of Parkinson’s disease patients (48, 49). For instance, the ceramide C24:1 (d18:1/24:1), which we found as one of the top elevated ceramides in the retina and RPE of *AdipoR1^–/–^* mice, was also identified as one of the 2 (next to d18:0/24:1) most highly elevated lipids in the primary visual cortex of Parkinson’s disease patients (50). The high level of C24:1 ceramide can be effectively lowered pharmacologically, as we showed in the DC-treated *AdipoR1^–/–^* mice.

The results described above warrant further investigation regarding the accumulation of aggregated protein in the photoreceptors, especially since it appears as one of the events leading to photoreceptor death, as suggested by our RNA-Seq analyses and also by another group (51).

### ADIPOR1 possesses intrinsic ceramidase activity, which is essential for healthy retinal function.

In direct biochemical assays, we confirmed that ADIPOR1 possesses ceramidase activity and demonstrated that adiponectin and C1Q increased the ADIPOR1 activity toward C18 hydrolysis by 24.7% and 34.4%, respectively (Figure 5G). Moreover, we showed that ablation of *AdipoR1* led to an approximately 22% reduction of the ocular ceramidase activity toward ceramide C24:1 (Figure 5F), which was also the highest elevated ceramide species in the RPE of *AdipoR1^–/–^* mice (Figure 5C). Our scRNA-Seq data indicate that *ADIPOR1* encodes the most abundantly expressed ceramidase in mice and one of the 2 most highly expressed ceramidases in the human retina, next to acid ceramidase *ASAH1* (Supplemental Figure 4). However, ASAH1 is a lysosomal ceramidase, while ADIPOR1 appears to be localized primarily in the outer cellular membrane; therefore, their ablation could generate different phenotypes. For instance, mice with *Asah1* deficiency also exhibited retinal pathology and impaired visual function, although the changes in retina structure were vastly different from those observed with *AdipoR1^–/–^* mice, with no photoreceptor degeneration in ~9-week-old mice but with severe retinal dysplasia and increased thickness of the ONL in those same mice (52). There were also not many microglia/macrophage cells detected in the retina of *Asah1^P361R/P361R^* mice, except for the optic nerve area. On the other hand, the profile of the accumulated ceramides in the retinas was somewhat similar to that for the retinas of *AdipoR1^–/–^* mice, with ceramides C24 and C22 being the most elevated species.

### Pharmacological lowering of ceramide generation increases photoreceptor survival and visual functions in AdipoR1^–/–^ mice.

Ceramide is considered to play a central role in sphingolipid metabolism (53). The metabolic pathways that lead to ceramide generation are the de novo synthesis pathway, the sphingomyelin hydrolysis pathway, or the salvage pathway in which complex sphingolipids are converted back to sphingosine and ceramide (Figure 9A). Increasing evidence suggests that elevated ceramide levels lead to photoreceptor degeneration, but overexpressing ceramidase or inhibiting ceramide generation pathways can rescue photoreceptor cells and prevent vision loss (15, 29, 45, 54–58). Therefore, to test whether we could increase photoreceptor survival in *AdipoR1^–/–^* mice, we decided to simultaneously inhibit 2 of the 3 ceramide generation routes, de novo biosynthesis and sphingomyelin hydrolysis. We used a combination of 2 drugs, DC, both previously shown to reduce ceramide levels. L-cycloserine is a widely used inhibitor of serine palmitoyl-CoA transferase, the rate-limiting enzyme that catalyzes the first step of ceramide de novo biosynthesis (28, 29, 59). Desipramine is a functional inhibitor of acidic sphingomyelinase (ASMase), which produces ceramide through hydrolysis of sphingomyelin (25, 27, 30, 39). By lowering ceramide levels in the retina with DC treatment, we reduced ceramide-driven photoreceptor apoptosis and increased the number of photoreceptor cells. Moreover, cone-mediated (photopic) retinal function in the DC-treated *AdipoR1^–/–^* mice also improved. Lastly, we found that prolonged DC-treatment corrected the electrical responses of the primary visual cortex to a visual stimulus, approaching near-normal levels for some parameters. These studies suggest that a similar approach could effectively attenuate the photoreceptor degeneration resulting from ADIPOR1 deficiency or elevated ceramide levels. Because noninvasive pharmacological intervention is more easily achieved in humans than gene therapy, the strategy proposed here might become applicable to humans in the long run, especially given that both drugs, DC, have been approved by FDA over 30 years ago and have been verified for safety. The drugs could be used in humans by oral administration, or — to avoid systemic toxicity at higher doses — they could be applied locally by continuous controlled release from an implanted intraocular capsule. However, future research should further develop and confirm these initial findings in animal and human trial studies to examine the efficacy and safety of the long-term usage of the combined DC therapy.

Taken together, our observations highlight the importance of ADIPOR1 as one of the principal ceramidases in the eye that regulate ceramide content, which is essential for healthy retinal function. They also show that ceramide accumulation can be effectively targeted pharmacologically by inhibiting 2 of the 3 ceramide generation pathways. These results provide a framework for developing new therapeutic strategies for treating retinal disorders and potentially other conditions caused by the pathologic build-up of ceramides, such as the ones observed in the *Abca4^−/−^* mouse model of Stargardt early-onset macular degeneration (30) or rd10 mouse model of RP (15).

## Methods

Supplemental Methods are available online with this article.

### Mice.

The mouse strain used for this research project, B6.129P2-*Adipor1^tm1Dgen^*/Mmnc, RRID: MMRRC_011599-UNC, was obtained from the Mutant Mouse Resource and Research Center (MMRRC) at the University of North Carolina at Chapel Hill, an NIH-funded strain repository; this strain was donated to the MMRRC by Deltagen. The obtained heterozygous mice were backcrossed into the C57BL/6J and B6(Cg)-*Tyr^c-2J^*/J backgrounds. Homozygous *AdipoR1^–/–^* mice were created by interbreeding of *AdipoR1^+/–^* mice. Animals were housed under 12-hour light/12-hour dark cycles and bred using standard procedures. Both male and female mice were used for all experiments.

### DC treatment.

Prior to the treatment, the dosing was chosen based on the toxicity studies in mice. We tested different doses of 3 drugs: desipramine (BML-AR119, Enzo Life Sciences), L-cycloserine (AK-87855, Ark Pharm), and fingolimod (FTY720; ceramide synthase inhibitor; S5002 Selleck Chemicals). The drug doses were as follows: (a) desipramine at 20, 40, 80 mg/kg; (b) L-cycloserine at 100, 200, and 400 mg/kg; and (c) FTY720 at 10, 20, and 40 mg/kg. Nine groups of mice (*n* = 2, each group) were given 1 of the 9 drug/dose combinations by i.p. injection every morning for 7 consecutive days. We monitored the animals’ weight and general condition. Out of 9 different conditions, we picked 2 that caused low toxicity (desipramine at 20 mg/kg and L-cycloserine at 200 mg/kg), and we used them both in a combined formulation.

Mice were injected i.p. 3 times a week for 20–22 weeks starting on P25, either with a drug cocktail containing 20 mg/kg desipramine and 200 mg/kg L-cycloserine dissolved in saline (DC-treated group) or with saline (control group). At P105, all mice were examined with OCT and SLO; at P109, all mice were examined with ERG. Finally, when they were 20–22 weeks old (at P140–P156), VEP were measured. After the VEP study, all mice were euthanized by CO_2_ inhalation, followed by cervical dislocation. Then, the left eye of each mouse was used for histology; the right eye was dissected to separate the retina from the RPE eyecup, and both tissues were analyzed for ceramide and sphingosine levels by LC-MS.

### Data availability.

Single-cell and bulk RNA-Seq data are deposited in the NCBI Gene Expression Omnibus under data repository accession no. GSE184902.

### Statistics.

Statistical analyses, other than those described above for visual cortex responses, were performed using GraphPad Prism software, and data are shown as mean ± SEM, unless otherwise stated. Results are considered significant if *P* was less than 0.05. The significance of differences between 2 groups was determined using a 2-tailed Student’s *t* test. For more than 2 groups, 1- or 2-way ANOVA was used with post hoc correction for multiple comparisons. When comparing multiple measurements within subjects, correction for repeated measures was performed

### Study approval.

All experiments were approved by the IACUC at the UCI (protocol no. AUP18-124), and were conducted following the ARVO Statement for the Use of Animals in Ophthalmic and Visual Research.

## Author contributions

DL and KP designed the study. DL, ATF, EHC, and KP designed experiments. DL, EHC, SS, HL, ZD, ET, J Luu, and XM performed experiments on mice. ATF performed visual cortex recordings. DL and RS produced and purified proteins. DL performed biochemical assays. JZ and AFMP performed LC-MS analyzes. MT analyzed RNA-Seq data with input from TH and AT. DL, ATF, EHC, SS, AFMP, ET, J Luu, J Lee, and DCL analyzed the data. EB contributed Abs. DL and KP supervised the project. DL prepared the figures with the help of ATF, TH, MT, DSK, and KP. DL wrote the manuscript with help from KP. SB, AS, DSK, and KP provided research resources. ATF, DCL, and DSK were involved in writing, and all authors contributed to editing and approved the manuscript.

## Supplementary Material

Supplemental data

## Version 1 01/11/2022

In-Press Preview

## Version 2 02/22/2022

Electronic publication
