# Supplementary material for: Inhibition of ceramide accumulation in AdipoR1–/– mice increases photoreceptor survival and improves vision
Source: JCI Insight. 2022 Feb 22;7(4):e156301. doi: 10.1172/jci.insight.156301 (PMC8876453; doi:10.1172/jci.insight.156301)
Supplement: Supplemental data [file jciinsight-7-156301-s091.pdf]

SUPPLEMENTAL MATERIAL

Inhibition of Ceramide Accumulation in *AdipoR1*$^{-/-}$ Mice Increases Photoreceptor Survival and Improves Vision

Dominik Lewandowski[1*], Andrzej Foik[2], Roman Smidak[1], Elliot H. Choi[1,3], Jianye Zhang[1], Thanh Hoang[4], Aleksander Tworak[1], Susie Suh[1,3], Henri Leinonen[1,5], Zhiqian Dong[6], Antonio F. M. Pinto[7], Emily Tom[1], Jennings Luu[1,3], Joan Lee[8], Xiuli Ma[6], Erhard Bieberich[9], Seth Blackshaw[4], Alan Saghatelian[7], David C. Lyon[10], Dorota Skowronska-Krawczyk[1,11], Marcin Tabaka[2], Krzysztof Palczewski[1,11,12,13*]

[1]Department of Ophthalmology, Gavin Herbert Eye Institute, UCI, Irvine, CA 92697, USA

[2]International Center for Translational Eye Research, Institute of Physical Chemistry, Polish Academy of Sciences, 01-224 Warsaw, Poland

[3]Medical Scientist Training Program, Case Western Reserve University, Cleveland, OH 44106, USA

[4]Solomon H. Snyder Department of Neuroscience, Johns Hopkins University, Baltimore, MD 21205, USA

[5]School of Pharmacy, University of Eastern Finland, 70211 Kuopio, Finland

[6]Polgenix Inc., Department of Medical Devices, Cleveland OH 44106, USA

[7]Clayton Foundation Laboratories for Peptide Biology, Salk Institute for Biological Studies, La Jolla, CA 92037, USA

[8]MetroHealth Medical Center, Case Western Reserve University, Cleveland, OH 44109, USA

[9]Department of Physiology, University of Kentucky, Lexington, KY 40536, USA

[10]Department of Anatomy and Neurobiology, UCI, Irvine, CA 92697, USA

[11]Department of Physiology and Biophysics, UCI, Irvine, Irvine, CA, USA

[12]Department of Chemistry, UCI, Irvine, CA 92697, USA

[13]Department of Molecular Biology and Biochemistry, UCI, Irvine, CA 92697, USA

*To whom correspondence should be addressed: Dominik Lewandowski, Department of Ophthalmology, Gavin Herbert Eye Institute, UCI, Irvine, CA 92697; lewandod@hs.uci.edu; Krzysztof Palczewski, Department of Ophthalmology, Gavin Herbert Eye Institute, UCI, Irvine, CA 92697; kpalczew@uci.edu; phone (949) 824-6527

Materials and Methods

Optical coherence tomography (OCT) and scanning laser ophthalmoscopy (SLO). Mice were anesthetized with a saline solution of ketamine/xylazine (100/10 mg/kg, i.p.), their pupils were dilated with 1% tropicamide and placed on a warming pad. OCT was performed with a Bioptigen spectral-domain OCT device (Leica Microsystems Inc., USA). Four frames of OCT b-scan images were acquired from a series of 1200 a-scans. Retinal ONL thickness was measured 500 μm away from the optic nerve head (ONH) in four retinal quadrants (nasal, temporal, superior, and inferior), using a ruler tool in ImageJ 1.52a software (NIH, USA). ONL thickness was averaged over the four retinal quadrants for all of the analyses. A blinded investigator analyzed OCT images from mice subjected to desipramine/L-cycloserine treatment on randomly coded images (Fig. 9C and S5A). SLO was performed to obtain whole-fundus images in vivo. Heidelberg Retinal Angiograph II (Franklin) in the autofluorescence mode was used to acquire the images, which were then analyzed qualitatively.

Hematoxylin and eosin (H&E) staining. Mice were euthanized by CO_2 inhalation followed by cervical dislocation. The superior side of the eyes was marked with a permanent blue marker, and the eyes were enucleated. Each eye was inserted into a tissue embedding cassette and transferred to Hartman's fixative (#H0290, Sigma Aldrich) for 24 hr at room temperature, then washed in 70% ethanol, embedded into paraffin, and sectioned at 7 μm thickness in the nasal-temporal orientation, yielding vertical, superior-inferior oriented sections. The sections were H&E-stained. Images were captured using a Keyence BZ-X800 microscope (Keyence Corp.) in the brightfield mode.

Electroretinography (ERG). Before recording, mice were dark-adapted for 24 hr. Under dim red light, mice were anesthetized with ketamine/xylazine (100/ 10 mg/kg, i.p.), their pupils were dilated with 1% tropicamide, and then 2.5% hypromellose (Akorn) was applied to keep the eyes hydrated. Contact lens electrodes were placed onto the corneas, and reference and ground electrodes were positioned subdermally between the ears and on the tail, respectively. ERGs shown in Fig. 2A, B, and S1 were recorded with the UTAS E-3000 system (LKC Technologies).

For single-flash scotopic ERG recording, the duration of white-light flash stimuli (from 20 µsec to 1 msec) was adjusted to provide a range of illumination intensities from −3.7 to 2.3 log ($cd \cdot s/m^2$). For each intensity, 3 to 20 recordings were made at sufficient intervals between flash stimuli (from 3 to 90 sec) to allow recovery from any photobleaching effects. The photopic ERG recordings were performed after bleaching at 1.4 log ($cd \cdot s/m^2$) for 10 min. For photopic ERG, the cone response was measured at four different light intensities (−0.7 to 2.3 log ($cd \cdot s/m^2$)) in the presence of rod-desensitizing white-light background.

For photopic ERG recordings shown in Fig. 11A and B, mice were light-adapted for 10 min before recording. Then, mice were anesthetized as described above, pupils were dilated with 1% tropicamide, and 2.5% hypromellose (Akorn) was applied. Active recording electrodes were placed onto the corneas, and reference and ground electrodes were positioned subdermally between the ears and on the tail, respectively. The eyes were stimulated with alternating blue and green stimuli at six different light intensities (0.1, 1, and 10 $cd \cdot s/m^2$ for blue, and 0.3, 3, and 30 $cd \cdot s/m^2$ for green). The ERGs were recorded with the Celeris rodent electrophysiology system (Diagnosys LLC) and analyzed with Espion V6 software (Diagnosys LLC). For all ERG comparisons, repeated measures two-way ANOVA followed by Sidak's post hoc test was used.

Retinoid Analyses were performed as previously described (1). Briefly, enucleated eyes of 24h-dark-adapted mice were deep-frozen in liquid nitrogen and stored at -80°C until analysis. All steps were performed in a dark room under dim red light. Frozen eyes were homogenized in 1 ml of retinoid analysis buffer (50 mM MOPS, 10 mM NH_4OH, and 50% ethanol in H_2O, pH 7.0). Retinoids were separated twice by centrifugation after adding 4 ml of hexane. Extracted retinoids in hexane were dried under a vacuum concentrator, suspended in 300 µl of hexane, and separated by normal-phase HPLC (Ultrasphere-Si, 4.6 × 250 mm; Beckman Coulter) in 10% ethyl acetate and 90% hexane (v/v) at a flow rate of 1.4 ml/min.

Western blots. Mice were euthanized by CO_2 inhalation followed by cervical dislocation. Retina and RPE-eyecups were dissected, followed by total protein extraction performed as described elsewhere (2). Briefly, retinas or RPE-eyecups from both eyes of the same mouse were pooled together and homogenized in lysis buffer composed of 1x RIPA buffer (#9806) with protease inhibitor cocktail (#04693132001, Roche). 200 µl of lysis buffer was added to the RPE-eyecup and retina samples; then, they were placed on ice for 20 min. Within that time, tubes with RPE-eyecups were frequently tapped over 50 times to dissociate RPE cells; and retina samples were homogenized for 30 sec using a tissue grinder (#12-141-361, Fisher Scientific). After 20 min, the remaining eyecups were removed, and the RPE cell suspension was vortexed briefly. All samples were sonicated for 3 cycles (2 sec ON/10 sec OFF) and centrifuged at 21,000 g for 15 min at 4°C. Supernatants were collected in new tubes, and protein concentration was measured using a BCA Assay Kit (#23227, Thermo Fisher). Before loading the gel, samples were prepared by mixing equal protein amounts (µg of total protein) of

individual samples with lysis buffer. Samples were subjected to SDS-PAGE electrophoresis, transferred to nitrocellulose membranes, and subsequently incubated for 1 hr at RT in tris-buffered saline containing 0.1% Tween20 (TBST) and 5% dry non-fat milk powder. One membrane was cut into two fragments (between the 37 kDa and 50 kDa protein standards), and the other identically prepared membrane was left intact. Membranes were incubated overnight at 4°C in TBST containing 2.5% dry non-fat milk powder with (i) ADIPOR1 Ab (1:200, #18993, IBL), or (ii) α-tubulin Ab (1:1000, #2144, Cell Signaling), used as a loading control. Membranes were washed 4x 5 min with TBST and incubated with HRP-labeled goat anti-rabbit Ab (1:3000, #7074, Cell Signaling) for 1 hr at RT. After washing (4 x 5 min with TBST), membranes were submerged in a chemiluminescent substrate (#34577, Thermo Fisher) for 5 min and imaged with Odyssey Fc System (LI-COR), which was also used for band quantification. Data were normalized to α-tubulin band intensities. The experiment was repeated two times.

Retinal and RPE-eyecup flatmounts. Mice were euthanized by CO_2 inhalation followed by cervical dislocation. The superior position of the eye was marked on the cornea with a blue Sharpie; globes were enucleated and fixed in PBS-buffered 4% paraformaldehyde (PFA) for 10 min. After fixing, eyes were washed three times in PBS for 3 min and placed on a microscope slide. Muscles, fat, and optic nerve were removed from the globe, followed by a puncture in the center of the cornea with a 25-gauge needle. Cohan-Vannas spring scissors (Fine Science Tools) were used to make four symmetric radial incisions starting from the center of the cornea and ending directly before the optic nerve head. The lens and vitreous were removed, and the eyecup was oriented with the sclera side facing up and flattened. The corneal flaps were removed, and the superior position was marked by making a small triangular cut. The RPE-eyecup was gently

peeled off the retina and flattened on a new slide, and the retina was flattened with the photoreceptors facing up. Next, both retina and RPE-eyecup were further fixed in PBS-buffered 4% PFA for 30 min. Finally, all flatmounts were washed three times in PBS for 5 min and used for immunofluorescence staining.

Retinal cross-sections. Mice were euthanized by CO_2 inhalation followed by cervical dislocation. Eyes were enucleated and rinsed briefly with PBS. Using a 23G needle, two small punctures were made in each eyeball at the junction of the cornea and sclera, and the eyes were fixed in PBS-buffered 4% PFA for 15 min. The cornea was cut off, followed by the lens and vitreous removal, and the eyecup was further fixed for 15 min. Next, the eyecup was gently washed three times in PBS for 5 min and transferred to a sucrose solution in PBS (10% and 20% sucrose for 30 min each at room temp., then 30% sucrose overnight at 4°C). On the next day, the eyecup was embedded in O.C.T. (Tissue-Tek) and snap-frozen in liquid nitrogen vapors. The eyecup was cut into 10 µm thick serial sections using a cryostat and stored at -80°C.

S-opsin and M-opsin staining. After fixation, retinal wholemounts were permeabilized with 0.5% Triton X-100 in PBS (PBST) for 30 min. Next, retinas floating in PBST were frozen at -80°C for 10 min and then thawed at room temperature to additionally increase tissue permeability. Thawed retinas were incubated for 3 nights at 4°C in goat polyclonal S-opsin Ab (#sc-14363, 1:4000 dilution; Santa Cruz Biotechnology) and rabbit polyclonal M-opsin Ab (#NB110-74730, 1:1000 dilution; Novus Biologicals) in PBST containing 5% normal donkey serum. After incubation with primary antibodies, retinas were washed three times for 10 min in PBST and then incubated for 2 hr at room temperature with secondary antibodies (#ab150129,

Abcam donkey anti-goat Alexa Fluor 488, and #ab150075, Abcam donkey anti-rabbit Alexa Fluor 647, at dilutions of 1:500 for both) in PBST. Retinas were washed three times for 10 min with PBST and then once with PBS. Finally, retinas were mounted with Vectashield medium (#H-1000; Vector Labs) and protected with coverslips. The experiment was repeated two times.

ADIPOR1 staining. Eye cross-sections were blocked and permeabilized with 10% normal goat serum (NGS; #ab7481, Abcam), 0.01% Triton X-100 in PBS for 1 hr at 22°C. Next, rabbit ADIPOR1 Ab was added at 1:100 dilution (#18993, IBL), together with PNA at 10 μg/ml (#FL-1071, Vector Labs) in PBS containing 10% NGS and 0.01% Triton X-100. Additionally, mouse 1d4 Ab (made in-house; (3)) was used for rhodopsin staining at 10 μg/ml. Sections were incubated overnight at 4°C. Next, after four rinses with PBS containing 0.01% Triton X-100, sections were incubated with a secondary goat anti-rabbit AF-647 (#A21245, ThermoFisher) at 1:1000 dilution in PBS containing 10% NGS and 0.01% Triton X-100, for 1 hr. To visualize rhodopsin-1d4 binding, goat anti-mouse AF-594 Ab (#A11032, ThermoFisher) was additionally applied. After five washes with PBS, sections were mounted with Vectashield medium with DAPI (#H-1500; Vector Labs). The experiment was repeated three times.

Iba1/F-actin staining. RPE-eyecup flatmounts were incubated in a blocking buffer consisting of 3% BSA with 0.3% Triton X-100 in PBS for 2 hr at 22°C. Next, rabbit Iba1 Ab was added at 1:1000 dilution (#019-19741, Fujifilm Wako) in blocking buffer overnight at 4°C, in a humidified chamber. Then the primary Ab was removed with five rinses of PBS with 0.3% Triton X-100. Flatmounts were incubated with a secondary goat anti-rabbit IgG Alexa Fluor 594 Ab (#A11037, Invitrogen) at 1:500 dilution in blocking buffer for 2 hr at 22°C, and then rinsed

four times with PBS containing 0.3% Triton X-100. The RPE-eyecup flatmounts were incubated in Phalloidin Alexa Fluor 488 (#A12379, ThermoFisher) at 1:1000 dilution in blocking buffer for 30 min at 22°C, and then rinsed four times with PBS with 0.3% Triton X-100 and once with PBS. Finally, specimens were mounted with Vectashield medium with DAPI (#H-2000; Vector Lab.) and protected with coverslips. Images were captured with a Z-stack mode using a Keyence BZ-X800 microscope (Keyence Corp.). The experiment was repeated two times.

Ceramide staining on RPE flatmounts. RPE-eyecup flatmounts were permeabilized with 0.2% Triton X-100 in PBS for 10 min, washed three times with PBS, and blocked with 100% BlockAid solution (#B10710, Invitrogen) for 2 hr at 22°C. Next, mouse ceramide Ab was added at 1:10 dilution (#ALX-804-196-T050, Enzo Life Sciences), with rabbit ZO-1 Ab at 1:100 dilution (#61-7300, Invitrogen) in 20% BlockAid overnight at 4°C. Then, the primary Ab was removed with five rinses of PBS. Flatmounts were incubated with a secondary goat anti-mouse Alexa Fluor 488 Ab (#A28175, Invitrogen) at 1:1000 dilution and goat anti-rabbit Alexa Fluor 647 Ab at 1:500 (#A21245, Invitrogen) in 20% BlockAid for 2 hr at 22°C, and then rinsed with PBS. Finally, specimens were mounted with Vectashield medium with DAPI (#H-2000; Vector Lab.) and protected with coverslips. The experiment was repeated two times.

Ceramide staining on eye cross-sections. Eye cross-sections were permeabilized with 0.2% Triton X-100 in PBS for 5 min, then washed four times with PBS and blocked with 0.5% ovalbumin in PBS for 1 hr at 22°C. Next, an unconjugated affinity-purified F(ab) fragment of anti-mouse IgG (#ab6668, Abcam) was added (0.1 mg/ml in PBS), incubated for 1 hr, and washed off with PBS. Subsequently, mouse ceramide Ab was added at 1:10 dilution (#ALX-804-

196-T050, Enzo Life Sciences), with PNA (10 μg/ml, #FL-1071, Vector Labs) in PBS containing 0.5% ovalbumin, and incubated overnight at 4°C. Next, after five rinses with PBS, sections were incubated for 1 hr with a secondary goat anti-mouse Ab AF-647 (#A21236, Life Technologies) at 1:1000 dilution in PBS containing 3% ovalbumin. After five washes with PBS, sections were mounted with Vectashield medium with DAPI (#H-1500; Vector Labs). The experiment was repeated two times.

TUNEL staining. TUNEL staining was performed as described by Histowiz Inc., using a Standard Operating Procedure and fully automated workflow. Samples were processed, embedded in paraffin, and sectioned at 4 μm. The TUNEL assay was developed using the DeadEnd Fluorometric TUNEL System (#G3250, Promega) according to the manufacturer's protocol and employing Bond Rx autostainer (Leica Biosystems). After staining, sections were dehydrated and coverslipped. Whole slide scanning (40x) was performed on an Aperio AT2 (Leica Biosystems).

Lipid extraction and LC-MS analysis – The data shown in Fig. 5A-C and Fig. S2 were generated at the MS Core at the Salk Institute. Lipids were extracted using a modified version of the Bligh-Dyer method (4). Briefly, tissue samples were homogenized with a probe sonicator in 1 mL of PBS. Samples were transferred to a glass vial (VWR), mixed with 1 mL methanol and 2 mL chloroform containing internal standards (^{13}C16-palmitic acid, d^{7}-Cholesterol), and shaken for 30 sec. The resulting mixture was vortexed for 15 sec and centrifuged at 2400 x g for 6 min to induce phase separation. The organic (bottom) layer was retrieved using a Pasteur pipette, dried under a gentle stream of nitrogen, and reconstituted in 2:1 chloroform:methanol for LC-MS

analysis. Lipidomic analysis was performed on a Vanquish HPLC online with a Q-Exactive quadrupole-orbitrap mass spectrometer equipped with an electrospray ion source (ThermoFisher). Data were acquired in positive and negative ionization modes. Solvent A consisted of 95:5 water:methanol, solvent B was 60:35:5 isopropanol:methanol:water. For positive mode, solvents A and B contained 5 mM ammonium formate with 0.1% formic acid; for negative mode, solvents contained 0.028% ammonium hydroxide. A Bio-Bond (Dikma) C4 column (5 µm, 4.6 mm × 50 mm) was used. The gradient was held at 0% B between 0 and 5 min, raised to 20% B at 5.1 min, increased linearly from 20% to 100% B between 5.1 and 55 min, held at 100% B between 55 min and 63 min, returned to 0% B at 63.1 min, and held at 0% B until 70 min. The flow rate was 0.1 mL/min from 0 to 5 min, 0.4 mL/min between 5.1 min and 55 min, and 0.5 mL/min between 55 and 70 min. Spray voltage was 3.5 kV and 2.5 kV for positive and negative ionization modes, respectively. Sheath, auxiliary, and sweep gases were 53, 14, and 3 units, respectively. The capillary temperature was 275°C. Data were collected in full MS/dd-MS2 mode (top 5). Full MS was acquired from 150–1500 m/z with a resolution of 70,000, AGC target of $1x10^6$, and a maximum injection time of 100 msec. MS2 was acquired with a resolution of 17,500, a fixed first mass of 50 m/z, AGC target of $1x10^5$, and a maximum injection time of 200 msec. Stepped normalized collision energies were 20, 30, and 40%.

Lipid identification was performed with LipidSearch (ThermoFisher). Mass accuracy, chromatography, and peak integration of all identified lipids were verified with Skyline (5). Skyline peak areas were used in data reporting, and data were normalized using internal standards and the protein content of the sample.

Ceramide and sphingoid base analyses shown in Fig. 10 and S6 were performed by the UCSD Lipidomics Core (www.ucsd-lipidmaps.org/home), using LC-MS according to validated procedures (6, 7).

Expression of ADIPOR1, H191A,H337A-mutant, adiponectin, and C1QTNF5. N-terminally truncated constructs of mouse ADIPOR1 (residues 89–375) and its H191A,H337A-mutant, both containing an N-terminal 6xHis-FLAG tag; full-length mouse adiponectin with C-terminal C-myc-6xHis tag, and full-length human C1QTNF5 with C-terminal FLAG-6xHis tag were cloned into pcDNA3.1 vectors (V79020, Invitrogen), and expressed in Expi293F GnTI$^-$ cells (#A39240, ThermoFisher). Cells were grown in suspension in Expi293 medium (#A1435101, Gibco) to a density of 2.5 x 10^6 cells/ml. Transfection mixtures were prepared by first diluting separately (i) 1.2 mg of plasmid DNA in 50 ml Opti-MEM (#11-058-021), and (ii) 3 mg of PEI MAX (#24765, Polysciences) in 50 ml Opti-MEM; then combining both mixtures, vortexing, incubating for 20 min, and adding dropwise to 900 ml of cell suspension. Cells producing membrane proteins were centrifuged 36 hr after transfection and stored at -80 °C until purification. Medium from cells producing adiponectin and C1QTNF5 was harvested on day 5 post-transfection, filtered, and stored until isolation at 4°C.

Purification of ADIPOR1 and the H191A,H337A-mutant. Thawed cells were lysed by osmotic shock in 10 mM Tris-HCl pH 7.5 with 2 mg/ml iodoacetamide (IAA) and protease inhibitors for 10 min and then centrifuged. The membrane proteins were extracted on ice using a dounce homogenizer in a buffer containing 20 mM HEPES (pH 7.5), 100 mM NaCl, 1% (w/v) n-dodecyl-β-d-maltoside (DDM), 0.1% (w/v) cholesteryl hemisuccinate (CHS; #D310-CH210,

Anatrace), 2 mg/ml IAA, and protease inhibitors. The extraction mixture was stirred for 15 min at 4°C, then centrifuged at 50,000 x g for 20 min, and cleared through a 0.45 μm syringe filter. The supernatant was adjusted to a final concentration of 20 mM HEPES (pH 7.4), 300 mM NaCl, 0.5% (w/v) DDM and 0.05% CHS and added to anti-FLAG magnetic agarose (#A36798 ThermoFisher). The resin was rotated 30 min at 4°C, washed six times in a buffer containing 20 mM HEPES (pH 7.4), 200 mM NaCl, 0.025% (w/v) DDM, and 0.025% (w/v) CHS, and the bound receptor was eluted in the same buffer supplemented with 2 mg/ml FLAG peptide, by rotating 1.5 hr at 4°C. The purity and concentration of the receptors were evaluated by SDS–PAGE and 660nm protein assay (#22660, ThermoFisher). After measuring the concentration, purified receptors were immediately used for the enzymatic assays.

Adiponectin and C1QTNF5 purification. Ni Sepharose (#17526801, Cytiva) was added to the medium containing expressed proteins and rotated for 2 hr at 4°C. The resin was collected and washed with buffers containing 25 mM HEPES pH 7.5, 300 mM NaCl, 1 mM $CaCl_2$, and increasing concentrations of imidazole (pH 7.5): 20 mM, 40 mM, and 60 mM. Finally, proteins were eluted in the same buffer containing 300 mM imidazole, dialyzed twice in a buffer containing 25 mM HEPES pH 7.5, 150 mM NaCl, 1 mM $CaCl_2$, and concentrated with Amicon Ultra-15 filters (#UFC903024, Millipore). Concentrated protein preparations were subjected to size-exclusion chromatography on a Superdex 200 Increase 10/300 GL (Cytiva) column. The obtained fractions of high-molecular-weight (HMW) adiponectin and C1QTNF5 were used in the functional assays.

Ceramidase assay of eye lysates. Enucleated mouse eyes from 20- to 25-day-old *AdipoR1*$^{+/+}$ and *AdipoR1*$^{-/-}$ mice were briefly rinsed with PBS, frozen on dry ice, and stored at -80°C until needed. Thawed eyes (n=8 for each genotype) were homogenized in 1.2 ml buffer containing 25 mM HEPES pH 7.5, 150 mM NaCl, 1 mM $CaCl_2$, 1% (w/v) DDM, and EDTA-free protease inhibitors; then sonicated on ice in five 2-sec cycles with 10-sec breaks, and spun for 15 min, 21,000 x *g*, 4°C. Supernatants were transferred to fresh tubes, and total protein concentration was measured by BCA assay. Supernatants were aliquoted in 1.5 ml tubes and incubated with 40 µM ceramide C2 (d18:1/2:0; #860502, Avanti Lipids), C18 (d18:1/18:0, #860518), or C24:1 (d18:1/24:1, #860525) for 3 hr at 37°C. The reaction was quenched by adding methanol (75% final), containing internal standard sphingosine-d7 (20 ng/ml final; # 860657, Avanti Lipids), and stored at -20°C until analysis. Sphingosine and sphingosine-d7 quantities were evaluated by LC-MS. The results were normalized to sphingosine-d7 and total protein concentration. The presented data are representative of three independently performed experiments. The experiment was repeated three times.

Sphingosine detection by LC-MS. Samples were spun (15 min, 21,000 x *g*, 22°C) and transferred to fresh tubes. Twenty microliters of the supernatant were injected into an Ultimate 3000 HPLC system coupled with an LXQ mass spectrometer (ThermoFisher) with an electrospray ionization unit. The separation was performed on a Proshell EC-18 column (2.7 µm, 3.0 x 150 mm, Agilent) using a mobile phase consisting of 0.1% aqueous formic acid (A) and acetonitrile (B) at a flow rate of 600 $\mu L \cdot min^{-1}$, and the mobile phase gradients and time course were as follows: 0-2 min, 2% B; 2-10 min, 100% B. The column chamber temperature was set at 30°C. The signals were detected in positive ionization multiple reaction monitor (MRM) mode.

The capillary temperature was set at 275°C, and the voltage was 5 kV. Sphingosine signals were monitored at the daughter ion of 282.3 (m/z) with the precursor of 300.3 (m/z), and d7-sphingosine signals were monitored at the 289.2 (m/z) fragment generated from the parent ion of 307.2 (m/z).

Ceramidase assay of purified ADIPOR1 and the H191A,H337A-mutant. Freshly purified ADIPOR1 and H191A,H337A-mutant (8 μM) were incubated with 30 μM ceramides C18 (18:1/18:0, #860518), or C24:1 (18:1/24:1, #860525) in a buffer containing 20 mM HEPES (pH 7.4), 150 mM NaCl, 0.025% (w/v) DDM and 1mM $CaCl_2$. Additionally, some samples were combined with purified proteins (5 μM): recombinant mouse HMW adiponectin, recombinant human HMW C1QTNF5, or native mouse C1Q (#M099, CompTech). The reactions were incubated for 3 hr at 37°C. The reactions were quenched by adding methanol (75% final) containing internal standard sphingosine-d7 (10 ng/ml final; # 860657, Avanti Lipids), and stored at -20°C until analysis. Quantities of sphingosine and sphingosine-d7 were evaluated by LC-MS. The results were normalized to sphingosine-d7. The presented data for enzymatic activity are representative of three experiments performed with three independent preparations of the ADIPOR1 WT and H191A,H337A-mutant proteins; each experiment comprised three or four replicates.

Bulk RNA-Seq of the retina and RPE. Eyes from *AdipoR1*$^{-/-}$ and WT mice at P30 were enucleated and placed in 60 mm dishes with PBS immediately following cervical dislocation. The anterior segment of the eye, including cornea, iris, and lens, was removed using dissecting scissors. The retinas from both eyes were carefully removed and transferred to 400 μl of

RNA*later* stabilization solution (#AM7920, Thermo Fisher). The posterior eyecups, consisting of RPE, choroid, and sclera, were transferred to 400 µl of RNAprotect tissue reagent (#76104, Qiagen). Eyecups were incubated at room temperature for 10 min and briefly agitated to detach RPE cells from the choroid/sclera. After removing the choroid-sclera, the remaining RPE cells were pelleted by centrifugation for 5 min at 685 x *g*. The isolated retinas and RPE cells in the stabilization solution were subjected to extraction of total RNA using the RNeasy Micro Kit (#74004, Qiagen) following the manufacturer's protocol.

RNA samples were processed in the Transcriptomics and Deep Sequencing Core (Johns Hopkins University) for library preparation and sequencing. Briefly, total RNA (~100 ng per sample) was used for library preparation by following the Illumina TruSeq Stranded Total RNA Library Prep Kit protocol. Libraries were then sequenced (paired-end 100 cycles) with the Illumina NextSeq 500 platform, yielding approximately 40 million raw reads per library.

Sequence data were demultiplexed using bcl2fastq software (Illumina), and their quality was assessed using FastQC (Babraham Bioinformatics). Reads were aligned to the reference mouse genome assembly (mm10) and quantified using the Rsubread software package with standard parameters (8), yielding on average 29 million counts per sample. Differential expression analysis was performed with EdgeR (v3.20.9)(9); and functional enrichment analysis was conducted on the identified differentially expressed genes ($log_2FC > 1$ & $FDR < 0.05$), using the findGO.pl function from the HOMER suite (Hypergeometric Optimization of Motif Enrichment, v4.9.1)(6). To correct for multiple hypotheses testing, the Benjamini and Hochberg FDR correction was used, and we retained terms at $FDR < 0.05$.

Single-cell RNA-Seq of *AdipoR1$^{-/-}$* and WT retinas. Eyes were enucleated for retinal tissue isolation. After removing the anterior chamber and lens, retinal cells were dissociated using the Papain Dissociation System (#LK003153, Worthington Biochemical) following the manufacturer's instructions. In each of the six experimental groups (WT and *AdipoR1$^{-/-}$* at P19, P25, and P30), four retinae were used to prepare single-cell suspensions at a final concentration of 1,000 cells/µl. For each group, freshly dissociated cells (~10,000) were loaded into a 10x Genomics Chromium Single Cell system using v2 chemistry following the manufacturer's instructions. In the first batch (B1: WT and *AdipoR1$^{-/-}$* at P30), two libraries were pooled and sequenced on Illumina NextSeq500 with ~76 million reads per library. In the second batch (B2: WT and *AdipoR1$^{-/-}$* at P19 and P25), four libraries were pooled and sequenced on Illumina NovaSeq6000 with ~143 million reads per library.

The preprocessing steps such as generation and demultiplexing of FASTQ files from the sequencing reads, aligning to UCSC mm10 transcriptome, and generating raw count matrices were conducted using Cell Ranger (v4.0.0 for B1, v6.0.1 for B2) (10x Genomics) with default parameters. Cumulus software v1.4.0 (10) was used to combine expression matrices and to remove cell doublets by Scrublet v0.2.1 (11). We performed downstream analysis using the R software package Seurat v3.2.2 (12). We removed low-quality cells with less than 500 genes detected and all genes expressed in less than 10 cells, leaving 26,764 cells and 19,324 genes for further analysis. Principal Component Analysis (PCA) was performed on a submatrix of the top 1,000 most variable genes computed, using the function *FindVariableGenes* from the Seurat package. Then, we removed the batch effect between samples using the package Harmony (13). In particular, we removed variables associated with two separate sequencing runs (B1 and B2) and WT and *AdipoR1$^{-/-}$* conditions to remove the non-cell-type-specific factors that impact cell clustering. We

evaluated the number of top principal components by the elbow method keeping 17 PCs for clustering and data visualization. The cells were clustered using a shared nearest neighbor (SNN) modularity optimization-based clustering algorithm (*FindClusters* in the Seurat package). To visualize cells in two dimensions, we used Uniform Manifold Approximation and Projection (UMAP) (14). The cluster-specific genes were computed using *FindAllMarkers* from the Seurat package, using the MAST test with the number of UMIs detected as a latent variable (15). The same test was used to compute differentially expressed genes between WT and *AdipoR1*$^{-/-}$ mice within cell clusters. Cell clusters were annotated by assessing known cell-type-specific markers. The gene set enrichment analysis was conducted as described in the bulk RNA-Seq data analysis section.

RNA-Seq data availability. Single-cell and bulk RNA-Seq data are deposited in the NCBI Gene Expression Omnibus under data repository accession number GSE184902.

Single-cell RNA-Seq of human and WT murine retinas shown in Fig. S4 was performed as previously described (16).

Single unit and local field potential recordings and visual stimulation. Mice were initially anesthetized with 2% isoflurane in a mixture of N_2O/O_2 (70%/30%), then placed into a stereotaxic apparatus. A small, custom-made plastic chamber was glued (3M Vetbond) to the exposed skull. After one day of recovery, re-anesthetized animals were placed in a custom-made hammock, maintained under isoflurane anesthesia (1-2% in a mixture of N_2O/O_2), and multiple tungsten electrodes were inserted into a small craniotomy above the visual cortex. Once the

electrodes were inserted, the chamber was filled with sterile agar and sealed with sterile bone wax. During recording sessions, animals were sedated with chlorprothixene hydrochloride (1 mg/kg; IM (17); and kept under light isoflurane anesthesia (0.2 – 0.4% in 30% O_2). EEG and EKG recordings were monitored throughout the experiments, and body temperature was maintained with a warming pad (Harvard Apparatus). Data were acquired using a 32-channel Scout recording system (Ripple, USA). The local field potential (LFP) from multiple locations was band-pass filtered from 0.1 Hz to 250 Hz and stored together with spiking data on a computer with a 1 kHz sampling rate. The LFP signal was cut according to stimulus time stamps and averaged across trials for each recording location to calculate visually evoked potentials (VEP)(18, 19). The spike signal was band-pass filtered from 500 Hz to 7 kHz and stored on a computer hard drive at 30 kHz sampling frequency. Spikes were sorted online in Trellis (Ripple, USA) during visual stimulation. Visual stimuli were generated in Matlab (Mathworks, USA) using the Psychophysics Toolbox (20, 21) and displayed on a gamma-corrected LCD monitor (55", 60 Hz; 1920 x 1080 pixels; 52 cd/m^2 mean luminance). Stimulus onset times were corrected for LCD monitor delay, using a photodiode and microcontroller (in-house design)(22).

The vision quality was assessed using a protocol published previously by our laboratories (19, 22-24). For recordings of visually evoked responses, cells were first tested with 100 repetitions of a 500 msec bright flash stimulus (105 cd/m^2). Receptive fields for visually responsive cells were then located using square-wave drifting gratings, after which optimal orientation/direction, and spatial and temporal frequencies were determined using sine-wave gratings. Spatial frequencies were from 0.001 to 0.5 cycles/deg. Temporal frequencies tested were 0.1 to 10 cycles/sec. With these optimal parameters, size tuning was assessed using apertures of 1 to 110° at 100% contrast. With the optimal size, temporal, and spatial frequencies,

and at high contrast, the orientation tuning of the cell was tested again using 8 orientations x 2 directions each, stepped by 22.5° increments. This was followed by testing the contrast response.

The LFP signal was normalized using z-score standardization (18, 25). The response amplitude of LFP was calculated as a difference between the peak of the positive and negative components in the VEP wave. The response latency was defined as the time point where maximum response occurred. The maximum response was defined as the maximum of either the negative or positive peak.

Tuning curves were calculated based on the average spike rate. Optimal visual parameters were chosen as the maximum response value.

The orientation tuning bandwidth was measured in degrees as the half-width at half-height (HWHH; 1.18 x σ) based on fits to Gaussian distributions (22, 26, 27), using:

$$R_{O_s} = baseline + R_p e^{-\frac{(O_s - O_p)^2}{2\sigma^2}} + R_n e^{-\frac{(O_s - O_p + 180)^2}{2\sigma^2}},$$

where O_s is the stimulus orientation, R_{Os} is the response to different orientations, O_p is the preferred orientation, R_p and R_n are the responses at the preferred and non-preferred directions, σ is the tuning width, and 'baseline' is the offset of the Gaussian distribution. Gaussian fits were estimated without subtracting spontaneous activity (26).

Size tuning curves were fitted by a difference of Gaussian (DoG) function:

$$R_s = K_e \int_{-s}^{s} e^{(-\frac{x}{r_e})^2} dx - K_i \int_{-s}^{s} e^{(-x/r_i)^2} dx + R_0,$$

where R_s is the response evoked by different aperture sizes. The free parameters, K_e and r_e, describe the strength and the size of the excitatory space, respectively; K_i and r_i represent the strength and the size of the inhibitory space, respectively; and R_0 is the spontaneous activity of the cell.

The optimal spatial and temporal frequency was extracted from the data fitted to Gaussian distributions using the following equation (22, 28):

$$R_{SF/TF} = baseline + R_{pref} e^{-\frac{(SF/TF - SF/TF_{pref})^2}{2\sigma^2}},$$

Where $R_{SF/TF}$ is the estimated response, R_{pref} indicates the response at the preferred spatial or temporal frequency, SF/TF indicates spatial or temporal frequency, σ is the standard deviation of the Gaussian, and baseline is the Gaussian offset.

The contrast tuning was fitted by using the Naka-Rushton equation (29):

$$R(C) = \frac{gC^n}{C_{50}^n + C^n},$$

where g is the gain (response), C_{50} is the contrast at mid response, and n is the exponent. For the contrast tuning fit, the background activity was subtracted from the response curve, and values below background standard deviation were changed to 0 (22, 30). Average differences between animal groups were considered statistically significant at $P \leq 0.05$ for two-tailed Mann-Whitney U-tests. Mean values given in the results include SEM. All offline data analysis and statistics were performed in Matlab.

Statistics. Statistical analyses, other than those described above for visual cortex responses, were performed using GraphPad Prism software, and data are shown as mean ± SEM, unless otherwise stated. Results are considered significant if *P* was less than 0.05. The significance of differences between 2 groups was determined using a 2-tailed Student's *t* test. For more than 2 groups, 1- or 2-way ANOVA was used with post hoc correction for multiple comparisons. When comparing multiple measurements within subjects, correction for repeated measures was performed.

Supplemental Figures

Content

Figure S1. ***AdipoR1*** **knockout alters retinal ERGs and retinoid cycle in mice.**
Figure S2. Ceramides accumulates in the retina and RPE-eyecup of ***AdipoR1***$^{-/-}$ **mice.**
Figure S3. Retinoids and A2E levels in the eyes of ***AdipoR1***$^{-/-}$**,** ***AdipoR1***$^{-/-}$***Rpe65***$^{-/-}$**,** ***AdipoR1***$^{-/-}$ ***Abca4***$^{-/-}$ **and** ***Abca4***$^{-/-}$***Rdh8***$^{-/-}$ **mice.**
Figure S4. Single-cell RNA-Seq analysis of the expression of genes involved in ceramide metabolism in the retina.
Figure S5. Intraperitoneal injection of desipramine/L-cycloserine (DC) cocktail delays photoreceptor death in ***AdipoR1***$^{-/-}$ **mice.**
Figure S6. Sphingosine levels in the eyes of ***AdipoR1***$^{-/-}$ **mice after 17-19-week treatment with saline or desipramine/L-cycloserine (DC) cocktail.**
Figure S7. Flash response from single neurons recorded in the primary visual cortex of untreated ***AdipoR1***$^{+/+}$ **mice and either saline- or DC-treated** ***AdipoR1***$^{-/-}$ **mice.**
Figure S8. Single-cell RNA-Seq analysis of ***AdipoR1***$^{-/-}$ **and WT retinas.**
Figure S9. Ceramidase activity of ADIPOR1.

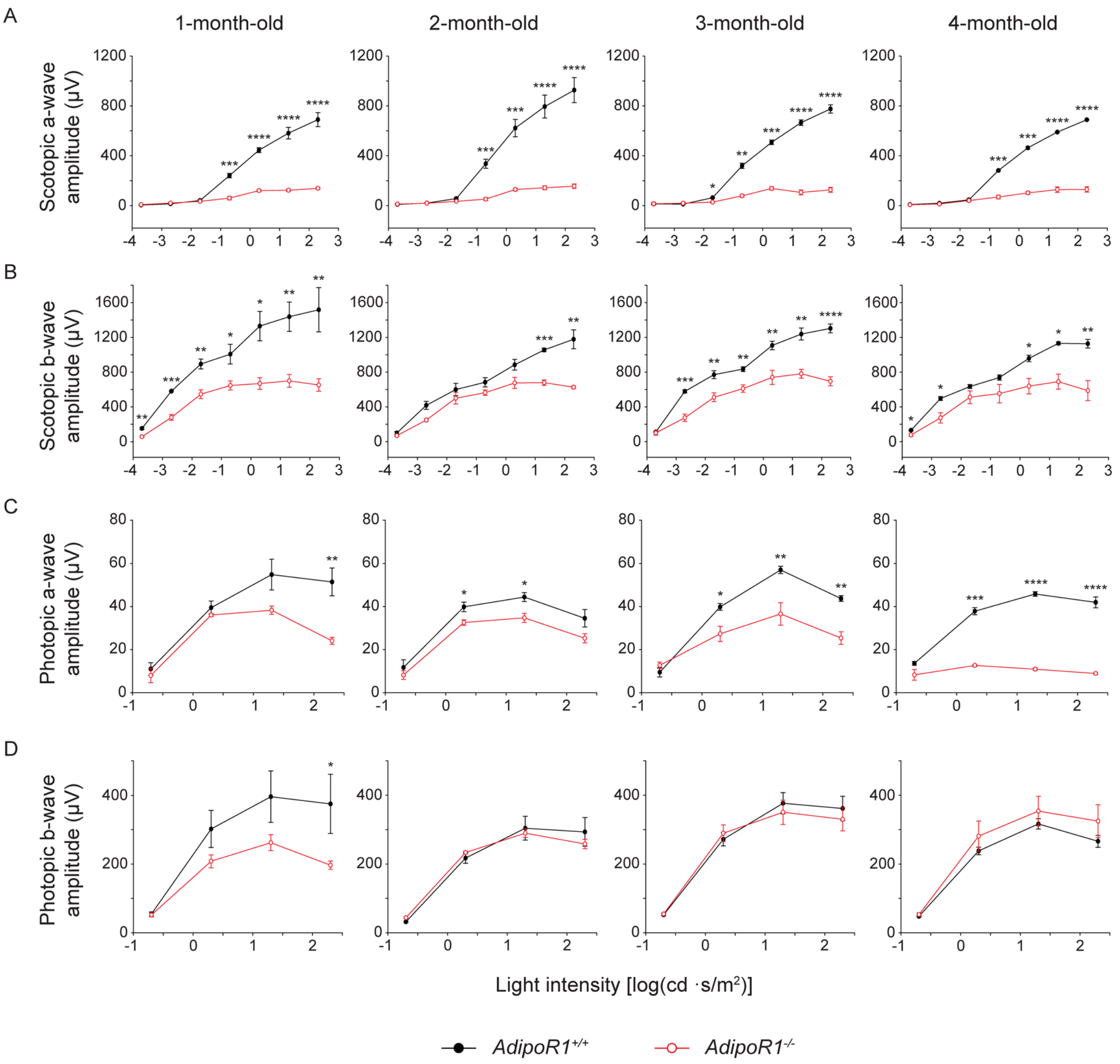

Figure S1. *AdipoR1* knockout alters retinal ERGs and retinoid cycle in mice. Serial responses to increasing flash stimuli were obtained for selected intensities under dark-adapted conditions (A, B); and under light-adapted conditions (C, D). Scotopic a-wave (A) and b-wave (B) amplitudes are dramatically attenuated in *AdipoR1*$^{-/-}$ mice. Photopic a-wave (C) and b-wave (D) amplitudes of *AdipoR1*$^{+/+}$ and *AdipoR1*$^{-/-}$ mice also show such attenuation, but the extent is animal-age sensitive ($n = 4$ for both genotypes and all ages). Repeated measures two-way ANOVA followed by Sidak's post hoc test was used; *$P < 0.05$, **$P < 0.01$, ***$P < 0.001$, ****$P < 0.0001$.

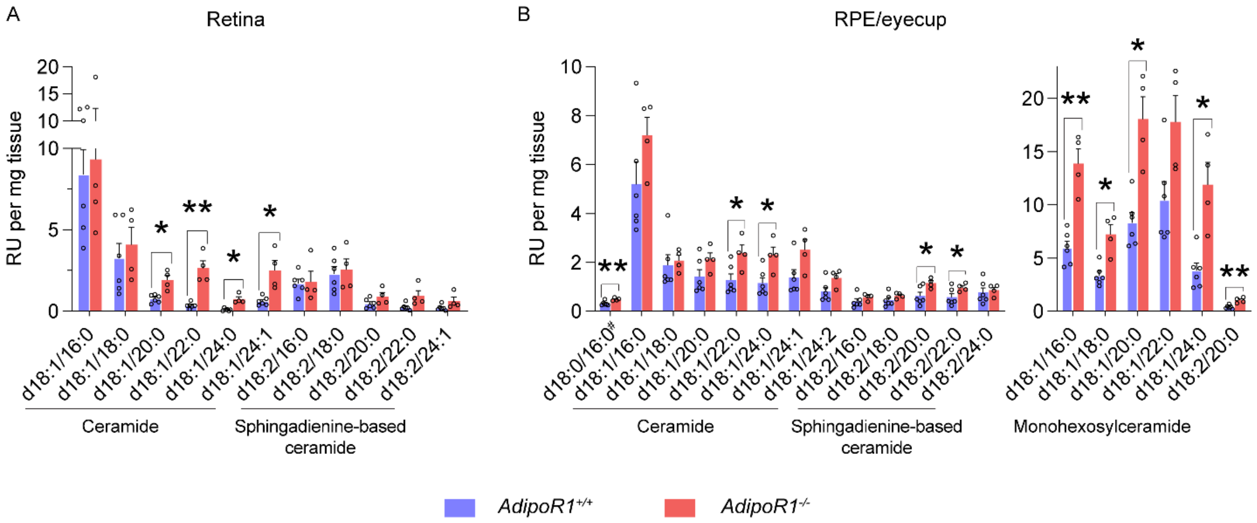

Figure S2. Ceramides accumulates in the retina and RPE-eyecup of *AdipoR1*$^{-/-}$ mice. Ceramide species detected in the (A) retina and (B) RPE-eyecup of 5-week-old *AdipoR1*$^{+/+}$ and *AdipoR1*$^{-/-}$ mice, quantified by LC-MS; # - dihydroceramide; n = 6 for *AdipoR1*$^{+/+}$ and n = 4 for *AdipoR1*$^{-/-}$ mice^{-}. Data represent mean ± SEM. The statistical significance was determined by two-tailed unequal variance (Welch) *t* test; *$P < 0.05$, **$P < 0.01$.

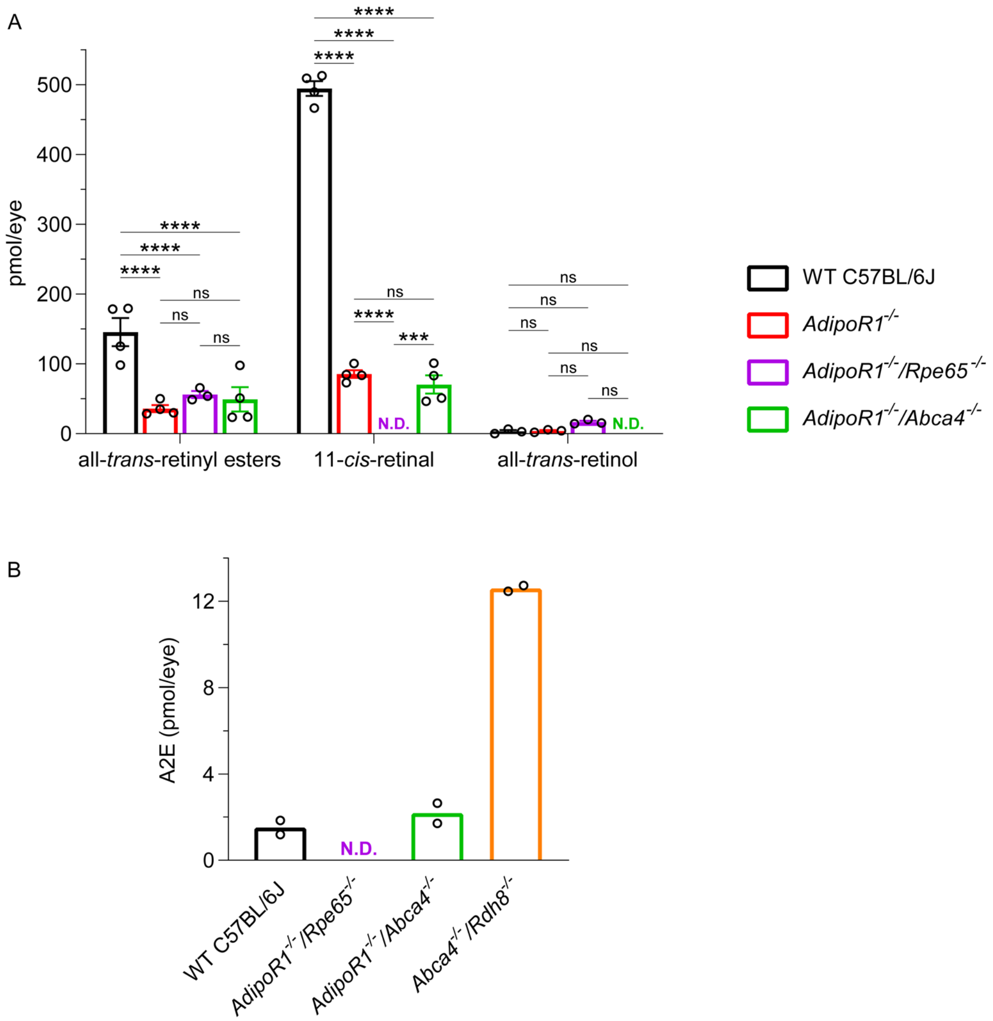

Figure S3. Retinoids and A2E levels in the eyes of *AdipoR1*$^{-/-}$, *AdipoR1*$^{-/-}$*Rpe65*$^{-/-}$, *AdipoR1*$^{-/-}$*Abca4*$^{-/-}$ and *Abca4*$^{-/-}$*Rdh8*$^{-/-}$ mice. (A) HPLC analysis of all-*trans*-retinyl esters, and levels of 11-*cis*-retinal and all-*trans*-retinol in the eyes of 3-month-old *AdipoR1*$^{-/-}$, *AdipoR1*$^{-/-}$*Rpe65*$^{-/-}$, and *AdipoR1*$^{-/-}$/*Abca4*$^{-/-}$ mice (n=4; for all-*trans*-retinol, n=3). (B) HPLC analysis of A2E levels in the eyes of 3-month-old WT, *AdipoR1*$^{-/-}$*Rpe65*$^{-/-}$, *AdipoR1*$^{-/-}$*Abca4*$^{-/-}$, and *Abca4*$^{-/-}$*Rdh8*$^{-/-}$ mice (n=2). The statistical significance was determined with two-way ANOVA followed by Tukey's multiple comparison test; *$P < 0.05$, **$P < 0.01$, ***$P < 0.001$, ****$P < 0.0001$.

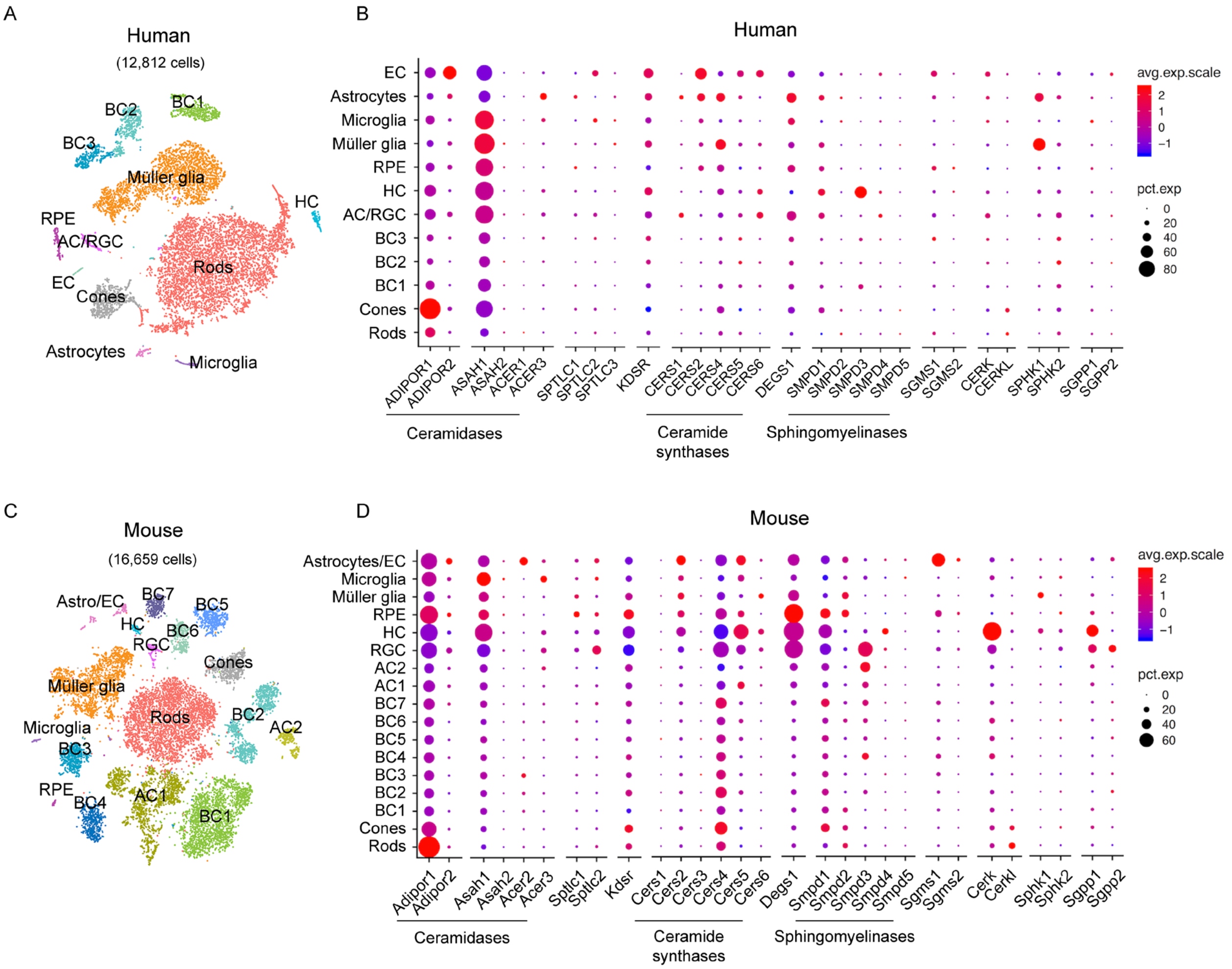

Figure S4. Single-cell RNA-Seq analysis of the expression of genes involved in ceramide metabolism in the retina. (A, C) tSNE view of retinal cells isolated from human (A) or mouse (C) origin that formed clusters representing each cell type. (B, D) Relative expression levels of genes involved in ceramide metabolism in various retinal cell types from humans (B) and mice (D). The dot size shows the percent of cells expressing the gene, and the color scale indicates the average expression level. EC, endothelial cells; RPE, retina pigment epithelium; HC, horizontal cells; RGC, retinal ganglion cells; AC, amacrine cells; BC, bipolar cells; SMC, smooth muscle cells.

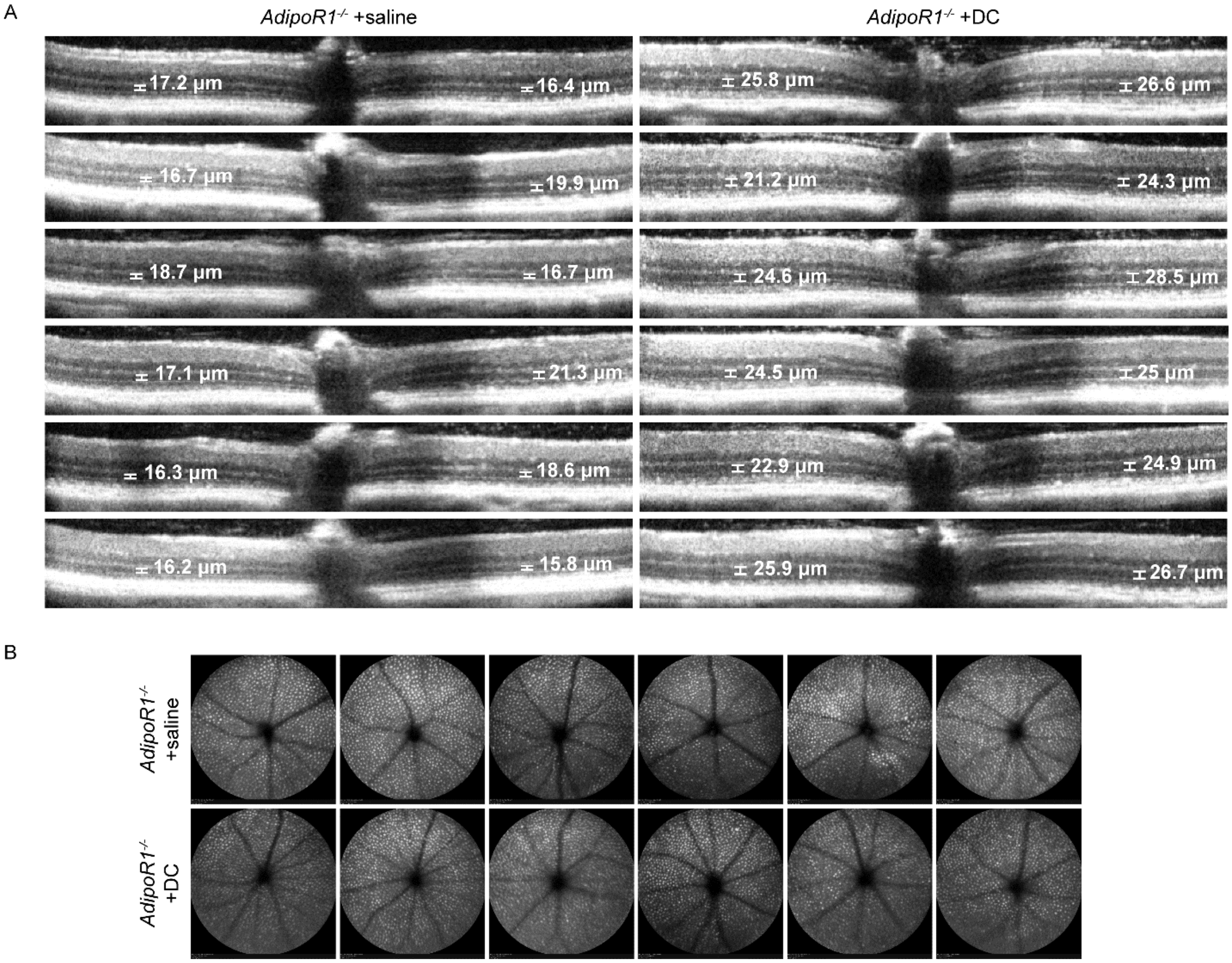

Figure S5. Intraperitoneal injection of desipramine/L-cycloserine (DC) cocktail delays photoreceptor death in *AdipoR1*$^{-/-}$ mice. (A) Representative OCT images of retinas from saline-treated and DC-treated *AdipoR1*$^{-/-}$ mice at P105 (after ~11-week treatment). White calipers at the inferior and superior aspects of the section indicate the ONL thickness measured 500 μm from the ONH; *n* = 6 for both groups. (B) Representative SLO images of eye fundi from saline-treated and DC-treated *AdipoR1*$^{-/-}$ mice at P105 (after ~11-week treatment); *n* = 6 for both groups.

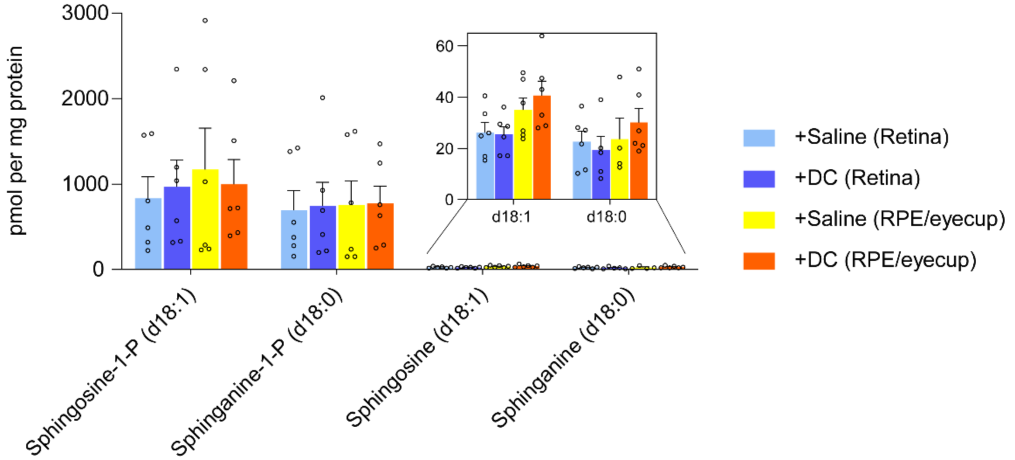

Figure S6. Sphingosine levels in the eyes of *AdipoR1*$^{-/-}$ mice after 17-19-week treatment with saline or desipramine/L-cycloserine (DC) cocktail. Two groups of *AdipoR1*$^{-/-}$ mice were injected i.p. with either saline or DC three times a week for ~17-19 weeks. After treatment, sphingosine levels in the retinas and RPE-eyecups were quantified by LC-MS. Data represent the mean ± SEM; n = 6 for both groups. Statistical significance was determined by a two-tailed unequal variance (Welch) t test, and no differences were found.

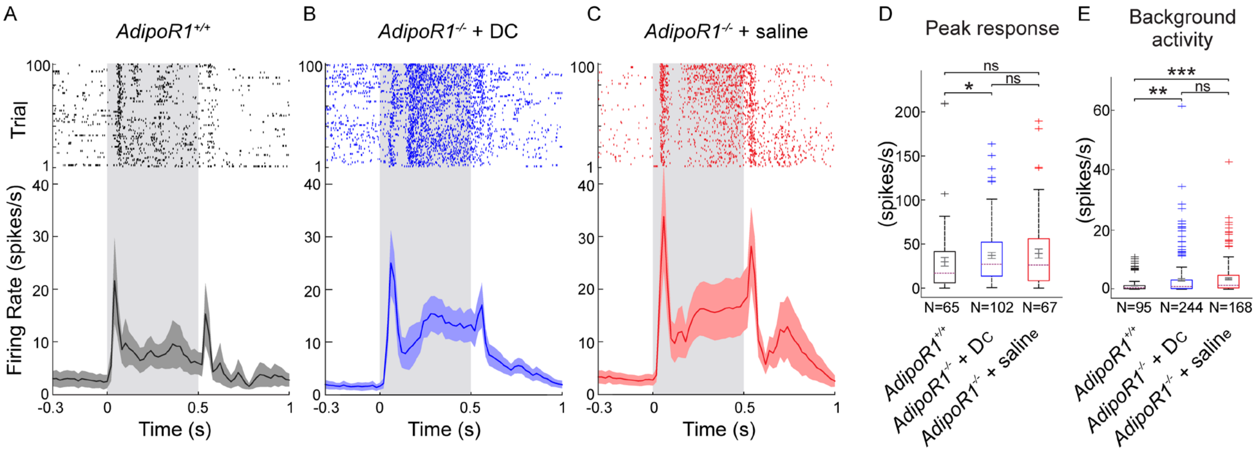

Figure S7. Flash response from single neurons recorded in the primary visual cortex of untreated *AdipoR1*$^{+/+}$ mice and either saline- or DC-treated *AdipoR1*$^{-/-}$ mice. Population histograms are shown of single-neuron flash responses recorded in *AdipoR1*$^{+/+}$ mice (A); *AdipoR1*$^{-/-}$ mice, treated with DC (B), or with saline (C), for ~17-19 weeks. The top portion of the graph in panels A-C is a raster plot of 100 rows of trials where each tic mark represents a single spike. (D) The maximum flash response rate is shown for each group. (E) The averaged spontaneous activity is shown for each group. For all panels, $n = 6$ for the *AdipoR1*$^{+/+}$group; and $n = 5$ for both *AdipoR1*$^{-/-}$ groups. "N" indicates the number of cells. Boxes extend from the 25th to the 75th percentiles. The dashed purple line indicates the median. Error bars inside the boxes indicate mean ± SEM. The whiskers extend to the most extreme data points that are not considered outliers; crosses beyond whiskers indicate outlying values. Statistical significance was determined with Kruskall-Wallis analysis followed by Dunn-Sidak multiple comparison correction; $*P < 0.05$, $**P < 0.01$, $***P < 0.001$, $****P < 0.001$.

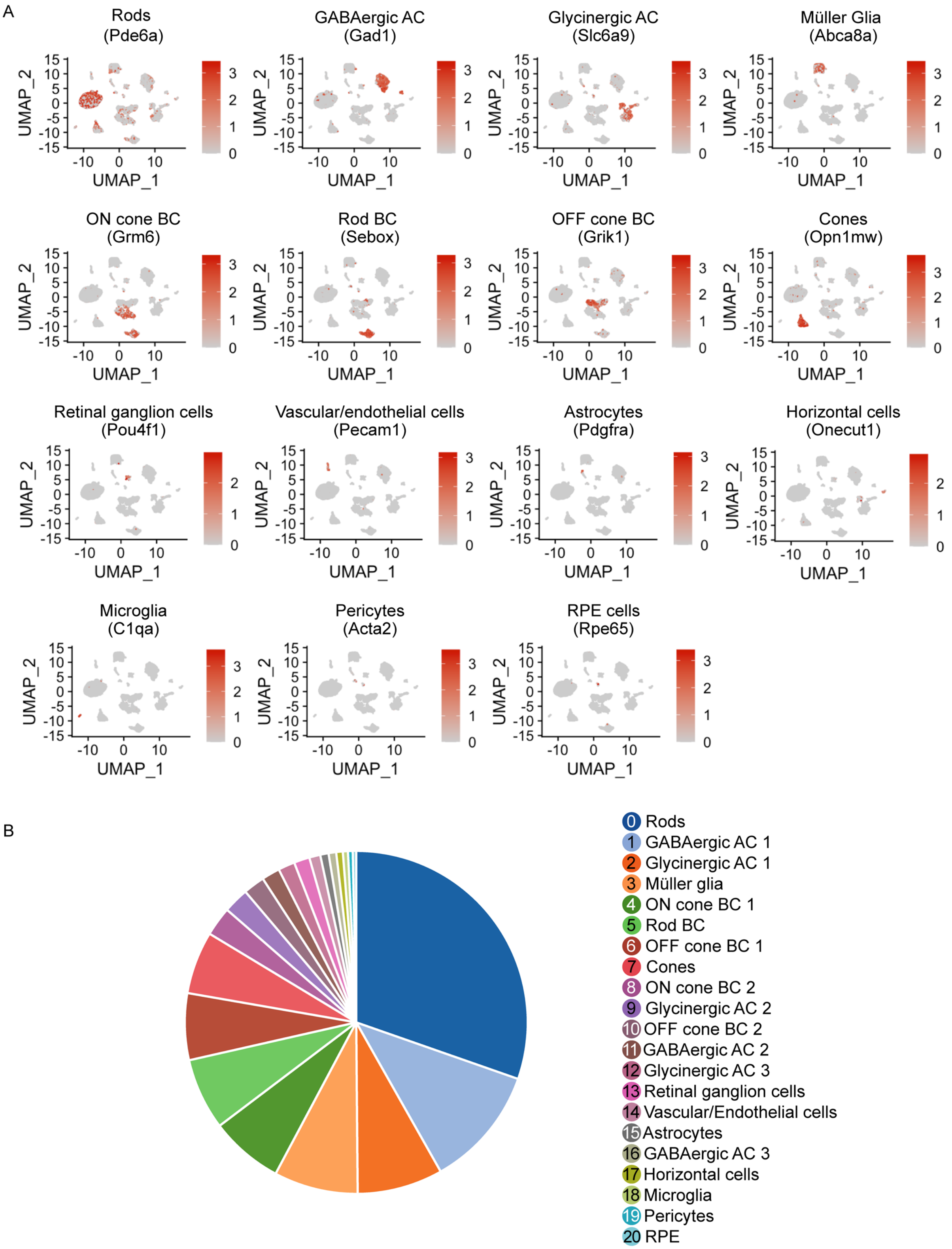

Figure S8. Single-cell RNA-Seq analysis of *AdipoR1*$^{-/-}$ and WT retinas. (A) UMAP plots are presented, showing the expression of mouse retinal cell markers used for cluster assignment. (B) A pie chart depicts the proportion of mouse retinal cell types identified in the scRNA-Seq

analysis of *AdipoR1*$^{-/-}$ and *AdipoR1*$^{+/+}$ at P19, P25, and P25. GABAergic – γ-aminobutyric acid-releasing; AC – amacrine cells; BC – bipolar cells.

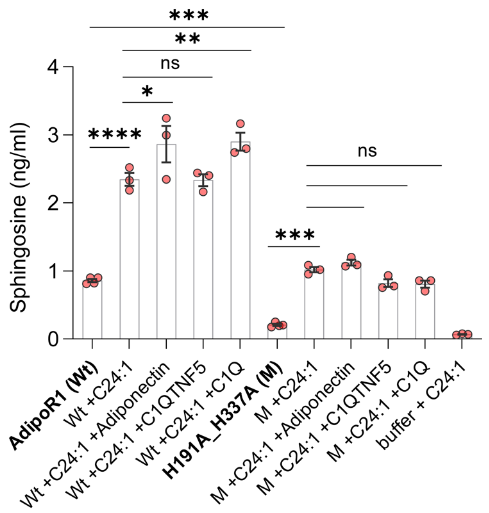

Figure S9. Ceramidase activity of ADIPOR1. The enzymatic assay was performed to determine the relative amounts of sphingosine produced by purified mouse ADIPOR1 wild type (WT) or H191A,H337A-mutant (M) forms of ADIPOR1, using ceramide C24:1 as substrate. The samples were additionally treated with high-molecular-weight fractions of recombinant mouse adiponectin, recombinant human C1QTNF5, or native mouse C1Q to examine their effect on the ceramidase activity of ADIPOR1. Detected sphingosine (d18:1) values were normalized to internal standard (sphingosine-d7). The data (mean ± SEM) are representative of three independent experiments performed in three or four replicates. Statistical significance was determined by one-way ANOVA followed by Sidak's post hoc test; $*P < 0.05$, $**P < 0.01$, $***P < 0.001$, $****P < 0.0001$.

References

1. Palczewska G, et al. Receptor MER Tyrosine Kinase Proto-oncogene (MERTK) Is Not Required for Transfer of Bis-retinoids to the Retinal Pigmented Epithelium. *J Biol Chem.* 2016;291(52):26937-49.
2. Wei H, et al. An easy, rapid method to isolate RPE cell protein from the mouse eye. *Exp Eye Res.* 2016;145:450-5.
3. MacKenzie D, et al. Localization of binding sites for carboxyl terminal specific anti-rhodopsin monoclonal antibodies using synthetic peptides. *Biochemistry-Us.* 1984;23(26):6544-9.
4. Bligh EG, and Dyer WJ. A rapid method of total lipid extraction and purification. *Can J Biochem Physiol.* 1959;37(8):911-7.
5. MacLean B, et al. Skyline: an open source document editor for creating and analyzing targeted proteomics experiments. *Bioinformatics.* 2010;26(7):966-8.
6. Hartler J, et al. Automated Annotation of Sphingolipids Including Accurate Identification of Hydroxylation Sites Using MSn Data. *Anal Chem.* 2020;92(20):14054-62.
7. Quehenberger O, et al. Lipidomics reveals a remarkable diversity of lipids in human plasma. *J Lipid Res.* 2010;51(11):3299-305.
8. Liao Y, Smyth GK, and Shi W. The R package Rsubread is easier, faster, cheaper and better for alignment and quantification of RNA sequencing reads. *Nucleic Acids Res.* 2019;47(8).
9. Robinson MD, McCarthy DJ, and Smyth GK. edgeR: a Bioconductor package for differential expression analysis of digital gene expression data. *Bioinformatics.*

2010;26(1):139-40.

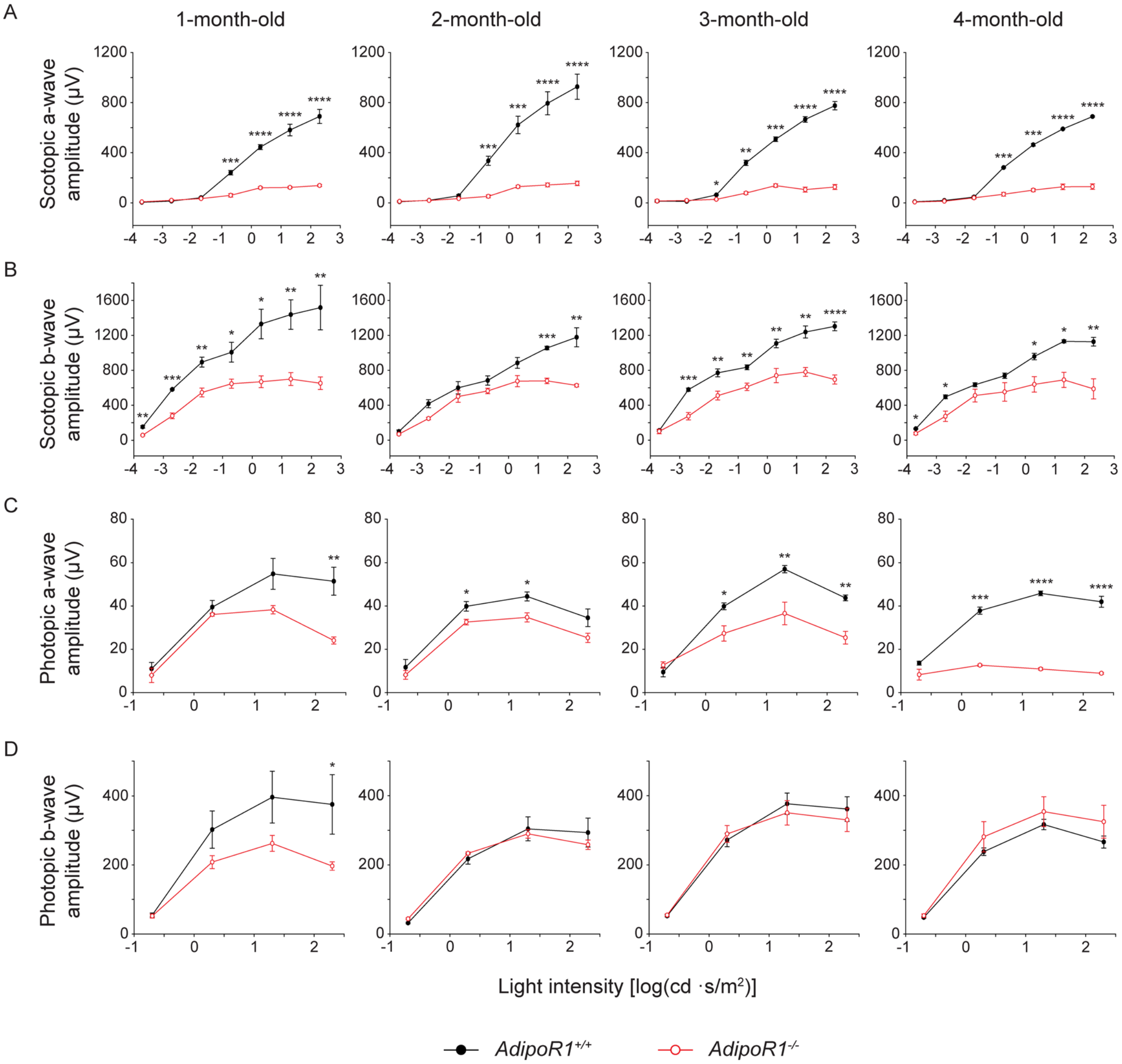

10. Li B, et al. Cumulus provides cloud-based data analysis for large-scale single-cell and single-nucleus RNA-seq. *Nat Methods.* 2020;17(8):793-+.

11. Wolock SL, Lopez R, and Klein AM. Scrublet: Computational Identification of Cell Doublets in Single-Cell Transcriptomic Data. *Cell Syst.* 2019;8(4):281-91 e9.

12. Butler A, et al. Integrating single-cell transcriptomic data across different conditions, technologies, and species. *Nat Biotechnol.* 2018;36(5):411-+.

13. Korsunsky I, et al. Fast, sensitive and accurate integration of single-cell data with Harmony. *Nat Methods.* 2019;16(12):1289-+.

14. Becht E, et al. Dimensionality reduction for visualizing single-cell data using UMAP. *Nat Biotechnol.* 2019;37(1):38-+.

15. Finak G, et al. MAST: a flexible statistical framework for assessing transcriptional changes and characterizing heterogeneity in single-cell RNA sequencing data. *Genome Biol.* 2015;16:278.

16. Zhang J, et al. Photic generation of 11-cis-retinal in bovine retinal pigment epithelium. *J Biol Chem.* 2019;294(50):19137-54.

17. Camillo D, et al. Visual Processing by Calretinin Expressing Inhibitory Neurons in Mouse Primary Visual Cortex. *Sci Rep.* 2018;8(1):12355.

18. Foik AT, et al. Retinal origin of electrically evoked potentials in response to transcorneal alternating current stimulation in the rat. *Invest Ophthalmol Vis Sci.* 2015;56(3):1711-8.

19. Suh S, et al. Restoration of visual function in adult mice with an inherited retinal disease via adenine base editing. *Nat Biomed Eng.* 2021;5(2):169-78.

20. Brainard DH. The Psychophysics Toolbox. *Spat Vis.* 1997;10(4):433-6.

21. Kleiner M, Brainard D, and Pelli D. What's new in Psychtoolbox-3? *Perception.* 2007;36:14-.

22. Foik AT, et al. Detailed Visual Cortical Responses Generated by Retinal Sheet Transplants in Rats with Severe Retinal Degeneration. *J Neurosci.* 2018;38(50):10709-24.

23. Kordecka K, et al. Cortical Inactivation Does Not Block Response Enhancement in the Superior Colliculus. *Front Syst Neurosci.* 2020;14.

24. Foik AT, et al. Visual Response Characteristics in Lateral and Medial Subdivisions of the Rat Pulvinar. *Neuroscience.* 2020;441:117-30.

25. Wypych M, et al. Standardized F1: a consistent measure of strength of modulation of visual responses to sine-wave drifting gratings. *Vision Res.* 2012;72:14-33.

26. Alitto HJ, and Usrey WM. Influence of contrast on orientation and temporal frequency tuning in ferret primary visual cortex. *J Neurophysiol.* 2004;91(6):2797-808.

27. Carandini M, and Ferster D. Membrane potential and firing rate in cat primary visual cortex. *J Neurosci.* 2000;20(1):470-84.

28. Deangelis GC, Ohzawa I, and Freeman RD. Spatiotemporal Organization of Simple-Cell Receptive-Fields in the Cats Striate Cortex .2. Linearity of Temporal and Spatial Summation. *J Neurophysiol.* 1993;69(4):1118-35.

29. Naka KI, and Rushton WA. S-potentials from luminosity units in the retina of fish (Cyprinidae). *J Physiol.* 1966;185(3):587-99.

30. Van den Bergh G, et al. Receptive-field properties of V1 and V2 neurons in mice and macaque monkeys. *J Comp Neurol.* 2010;518(11):2051-70.
